# Autophagy Suppresses RIP Kinase-Dependent Necrosis Enabling Survival to mTOR Inhibition

**DOI:** 10.1371/journal.pone.0041831

**Published:** 2012-07-26

**Authors:** Kevin Bray, Robin Mathew, Alexandria Lau, Jurre J. Kamphorst, Jing Fan, Jim Chen, Hsin-Yi Chen, Anahita Ghavami, Mark Stein, Robert S. DiPaola, Donna Zhang, Joshua D. Rabinowitz, Eileen White

**Affiliations:** 1 The Cancer Institute of New Jersey, New Brunswick, New Jersey, United States of America; 2 University of Medicine and Dentistry of New Jersey, Piscataway, New Jersey, United States of America; 3 Department of Pharmacology and Toxicology, University of Arizona, Tucson, Arizona, United States of America; 4 Division of Medical Oncology, University of Medicine and Dentistry of New Jersey, Piscataway, New Jersey, United States of America; 5 Department of Molecular Biology and Biochemistry, Rutgers University, Piscataway, New Jersey, United States of America; 6 Department of Chemistry, Lewis-Sigler Institute for Integrative Genomics, Princeton University, Princeton, New Jersey, United States of America; Istituto Nazionale per le Malattie Infettive, Italy

## Abstract

mTOR inhibitors are used clinically to treat renal cancer but are not curative. Here we show that autophagy is a resistance mechanism of human renal cell carcinoma (RCC) cell lines to mTOR inhibitors. RCC cell lines have high basal autophagy that is required for survival to mTOR inhibition. In RCC4 cells, inhibition of mTOR with CCI-779 stimulates autophagy and eliminates RIP kinases (RIPKs) and this is blocked by autophagy inhibition, which induces RIPK- and ROS-dependent necroptosis *in vitro* and suppresses xenograft growth. Autophagy of mitochondria is required for cell survival since mTOR inhibition turns off Nrf2 antioxidant defense. Thus, coordinate mTOR and autophagy inhibition leads to an imbalance between ROS production and defense, causing necroptosis that may enhance cancer treatment efficacy.

## Introduction

Macroautophagy (autophagy) is a process whereby cellular components such as proteins and organelles are captured within autophagosomes, and are delivered to the lysosomes for degradation [1]. Autophagy is activated by starvation, enabling cellular and mammalian viability by providing an internal source of building blocks for macromolecular synthesis. This sustains biosynthetic and energy requirements during interruptions in nutrient availability [2], [3], [4], [5]. Cellular damage also activates autophagy, which promotes cell survival by preventing accumulation of damaged proteins and organelles, particularly mitochondria that are a source of oxidative stress [6], [7]. These intracellular recycling and garbage disposal roles of autophagy provide cells with the inherent flexibility to cope with periods of deprivation while limiting oxidative stress. Although autophagy is important for the function and survival of normal cells, it is also utilized by tumor cells and may be therapeutically counterproductive [5], [8], [9], [10], [11].

Metabolic stress due to oxygen, nutrient and growth factor deprivation is common in tumors due to insufficient vascularization, and autophagy is activated in tumor cells in hypoxic regions to support survival [12], [13], [14]. Tumor cells in oxygen-deprived hypoxic regions are more resistant to treatment and this autophagy-conferred survival advantage has led to the realization that autophagy inhibitors may be useful to improve cancer therapy [15], [16]. Moreover, cytotoxic and targeted cancer therapeutics induce autophagy, either by infliction of stress and cellular damage or by inhibition of signaling pathways mimicking deprivation [5], [15]. The potential of environmental and therapeutic autophagy induction to limit treatment effectiveness remains to be addressed.

Cells respond to growth signals in their environment through the phosphoinositide 3-kinase (PI3K) pathway and regulation of the serine-threonine kinase mTOR. mTOR promotes cell growth in response to nutrient and growth factor availability, while suppressing autophagy [17]. In nutrient and growth factor replete conditions, the PI3K pathway activates mTOR through the mTORC1 complex that binds, phosphorylates and inhibits key autophagy machinery components required to initiate autophagosome formation [18], [19], [20], [21]. Tumor cells commonly take advantage of the cell growth-promoting function of the PI3K pathway by acquiring mutations that result in its constitutive activation [17]. As such, inhibitors of the PI3K pathway are potentially useful for restricting tumor growth, although they also activate autophagy. It is unclear, however, if this induction of autophagy is a mechanism of resistance by increasing tumor cell stress tolerance.

mTOR inhibitors include both allosteric (rapamycin/sirolimus, and other rapalogs such as temsirolimus and everolimus) and ATP-competitive mechanistic classes. Both temsirolimus and everolimus have shown efficacy in the treatment of renal cancer and are approved by the Food and Drug Administration (FDA) to treat RCC [22], [23]. In the first line setting, temsirolimus improves overall survival in RCC patients with metastatic disease. Unfortunately, all patients eventually relapse, contributing to the dismal prognosis of this devastating disease. It is expected that most targeted therapeutics, including temsirolimus, will require combination therapy for improved therapeutic outcome, necessitating identification of synthetic lethal pathways.

If autophagy induction by mTOR inhibitors promotes tumor cell survival, then combination treatment with an autophagy inhibitor may be expected to promote tumor cell death [11]. One tractable, small molecule approach to autophagy inhibition is the lysosomotropic anti-malaria drug CQ and its analogues. CQ prevents lysosome acidification and thereby the degradation of the products of autophagy, resulting in autophagolysosome accumulation. In a mouse model of c-Myc-driven lymphoma, a CQ analogue hydroxychloroquine (HCQ) promotes tumor cell death by either p53 activation or alkylating agents [24]. In mouse models for ataxia telangiectasia and Burkitt's lymphoma, CQ suppresses spontaneous tumorigenesis [25]. CQ also has antitumor activity in mouse models of BRC-Abl leukemia [26] and in combination with the HDAC inhibitor suberoylanilide hydroxamic acid (SAHA) in a mouse model and in human samples of CML [27]. In human solid tumor xenografts, CQ inhibits tumor growth as a single agent in pancreatic cancer, and in combination with PI3K pathway inhibitors in glioma or with mTOR inhibitors in tuberous sclerosis complex (TSC)-deficient tumors [28], [29], [30]. These preclinical studies support the approach of autophagy inhibition in cancer therapy [15], [16]. One major limitation is the unknown mechanism by which autophagy inhibition promotes cancer cell death, particularly in combination with PI3K pathway inhibition, and what defines cancer susceptibility to autophagy inhibition.

Since the mTOR inhibitor temsirolimus (Torisel or CCI-779) has efficacy in RCC [23] and promotes autophagy [31], [32], we assessed the functional consequences of coordinate autophagy inhibition with CQ in human RCC cell lines. We found that mTOR inhibition induced selective autophagy of mitochondria (mitophagy) and eliminated RIPKs, the master regulators of necroptosis. Addition of CQ promoted cell death by preventing autophagy, causing RIPK- and ROS-mediated necroptosis. As mTOR inhibition blocked Nrf2 nuclear translocation and antioxidant defense by activating GSK3β, RCC cell lines were sensitized to inhibition of autophagy with CQ and the subsequent ROS production. Thus, autophagy is a cancer therapy survival mechanism to RIPK-dependent necroptosis in the setting of cancer therapy. These findings support clinical assessment of autophagy inhibition to augment the activity of mTOR inhibitors in RCC where Nrf2, autophagy, and RIPK elimination are required for survival.

## Results

### Human RCC lines have high levels of basal autophagy

**Figure 1:**
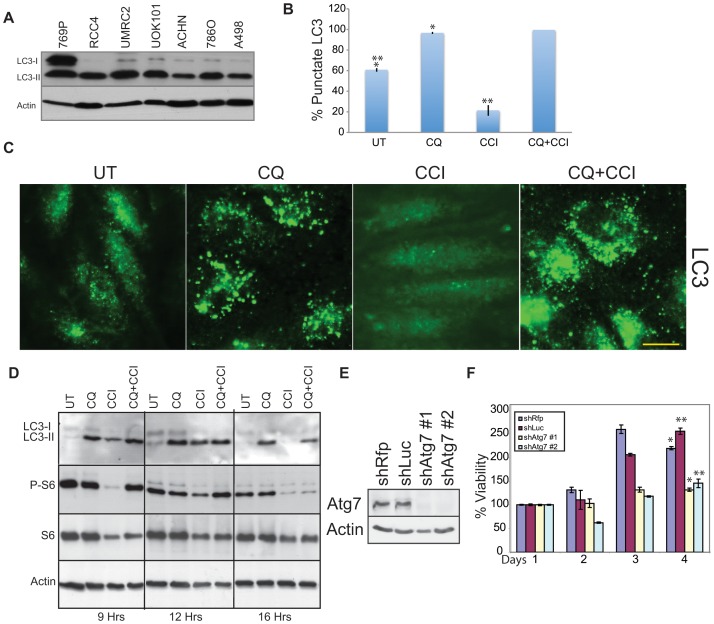
The human renal cancer cell line RCC4 has high basal autophagy. (A) LC3 immunoblotting in human RCC cell lines. Human RCC cells have high basal autophagy as indicated by the presence of the lipidated-conjugate, LC3-II, under normal culture conditions. (B) Quantitation of LC3 punctation in RCC4 under a 9 hour drug treatment. 40 µM CQ caused accumulation of autophagosomes above basal levels (*p = 3.05×10^−5^) while 40 µM CCI-779 caused clearance of autophagosomes after a 9 hour drug treatment (**p = 0.0013). Error bars represent ± standard deviation (S.D.). (C) LC3 immunostaining of RCC4. Representative photos of Figure 1B. Scale bar = 10 microns. (D) Western blot of RCC4 showing accumulation of LC3-II with CQ and clearance of LC3-II with CCI-779 at 9, 12 and 16 hours. S6 lost phosphorylation with CCI-779 treatment while total S6 level remained the same. (E) Western blot showing knockdown of Atg7 in RCC4 after expression of two shRNAs targeted against Atg7 (sh*atg*7 #1, sh*atg*7 #2), as compared to control siRNA's, shRfps or shLUC. (F) Viability graph showing impaired viability and cell growth after *atg7* knockdown. Error bars represent ± standard deviation (S.D.). (*p = 0.030 **p = 0.002).

Human RCCs have high basal autophagy as indicated by processing of LC3-I to the autophagosome localized LC3-II form (Figure 1A) and 30–60% LC3 punctation (Figure S1A) that are substantially higher than the 1–5% level in normal kidney cell lines [14]. All cell lines display 100% LC3 punctation when treated with CQ (Figure S1A). In RCC4, high basal autophagy occurred despite presence of active PI3K/mTOR signaling (phosphorylation S6, (Figure 1D). CQ caused accumulation of LC3 puncta and LC3-II indicating high flux through the autophagy pathway and that autophagy was blocked (Figure 1B–D). The mTOR inhibitor CCI-779 blocked phosphorylation of S6, indicating that mTOR kinase activity was inhibited (Figure 1D). CQ partially rescued this at 9 hours, which may result from perturbation of lysosomes. CCI-779 increased turnover and clearance of LC3 positive autophagosomes and LC3-II by Western blot (Figure 1D) indicating increased autophagic flux, which was blocked with CQ (Figure 1B–C). Knockdown of the essential autophagy gene product Atg7 with 2 independent shRNAs impaired growth in RCC4 cells as compared to two control hairpins (Figure 1E and 1F). Thus, RCC cells have high basal autophagy that was required for growth, stimulated by CCI-779, blocked by CQ and consistent with autophagy addiction commonly found in cancer cell lines [4], [29], [33]

### Cytotoxicity of coordinate mTOR and autophagy inhibition

**Figure 2:**
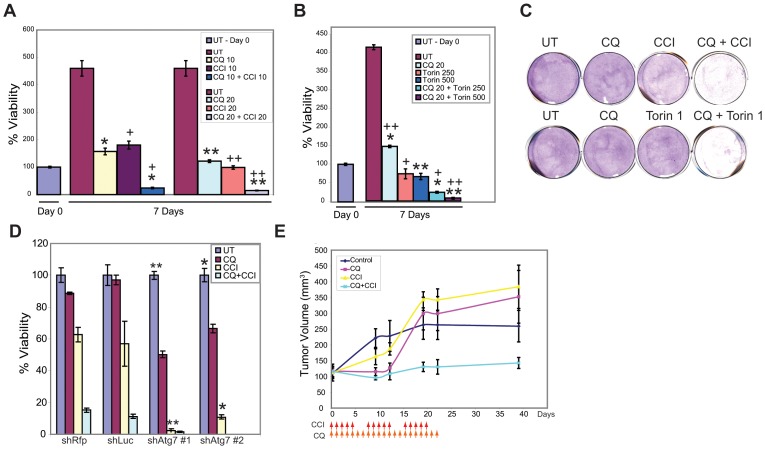
Coordinate autophagy and mTOR inhibition is cytotoxic to human RCC. (A) Viability graph of RCC4 cells treated with 10 µM or 20 µM CQ, 20 µM CCI-779 or combination of CQ and CCI-779. After 7 days, cells treated with CQ and CCI-779 as single agents were viable and showed growth arrest. The combination of 20 µM CQ with 20 µM CCI-779 reduced viability to 10%. (*p = 0.033, +p = 0.038,**p = 0.016,++p = 0.028.) (B) Viability graph of 20 µM CQ alone, 250 nM or 500 nM Torin1 or the combination of CQ and Torin1. After 7 days CQ treated cells were viable, Torin1 treated cells were 60% viable, and the combination of CQ and Torin1 were 20% viable (20 µM CQ+250 nM Torin1) and 10% viable (20 µM CQ+500 nM Torin1). Viability was measured by trypan blue exclusion and is relative to day 0. Error bars represent ± S.D. (*p = 0.020,+p = 0.036,**0.016, (C) Clonogenic survival assay showing that the combination of CQ and CCI-779 or CQ and Torin1 severely impairs long term survival. (D) % viability of knockdown cells under 40 µM CQ, 40 µM CCI-779 or combination of CQ and CCI-779 for 18 hours. CCI-779 alone with *atg7* knockdown induced significant cell death (*p = 0.002 **p = 2.01×10^−4^). Error bars represent ± S.D. (E). RCC4 cells were grown as xenografts in nude mice. When tumors reached 150 mm^3^, mice were injected with vehicle (dark blue), CQ (red), CCI-779 (yellow) or combination of CQ and CCI-779 (light blue). Combination of CQ and CCI-779 prevented further tumor growth as compared to CQ alone (p = 0.011) or CCI-779 (p = 0.003). Arrows indicate days that CQ and CCI-779 were injected. Error bars are ± s.e.m.

To test if mTOR inhibitors promote survival by autophagy, RCC4 was treated with allosteric (CCI-779), or catalytic (Torin1), mTOR inhibitors alone or in combination with CQ. CQ, CCI-779 and Torin1 were predominantly cytostatic, but either mTOR inhibitor was cytotoxic when combined with CQ (Figure 2A, 2B, S2A). After 5 days, cells recovered without drugs and clonogenic potential was assessed. CQ, CCI-779 and Torin1 had no effect on recoverable growth, while the combination of CQ with CCI-779 or Torin1 prevented clonogenic survival (Figure 2C). The combination of CQ and CCI-779 was equally cytotoxic to VHL-mutant RCC cell lines (RCC4, A498) and to these cell lines that had VHL reexpressed, suggesting that sensitivity was HIF-independent (Figure S1B, and Movies S1, S2, S3, S4, S5, S6, S7, S8, S9, S10, S11, S12, S13, S14, S15, and S16). Knockdown of Atg7 with 2 different shRNAs also sensitized RCC4 cells to CCI-779, indicating that autophagy was responsible for resistance to mTOR inhibition (Figure 2D and S2B). To determine if the combination of CQ and CCI-779 could suppress tumor growth, RCC4 xenografts were examined. Tumors continued to grow when treated with vehicle, CQ or CCI-779, while the combination of CQ and CCI-779 prevented tumor growth (Figure 2E). Importantly, mice appeared healthy during of the treatment with no observable weight loss or change in behavior indicating that the treatment was well tolerated. Thus, autophagy is a resistance mechanism to CCI-779 that could be reversed by genetic blockade of autophagy initiation or pharmacologic blockade of cargo degradation.

### mTOR inhibition and CQ induce RIPK -dependent necroptosis

To determine the type of cell death induced by the combination of CQ and CCI-779, RCC4 cells were examined for cleavage of the apoptosis marker caspase-3. Minimal active caspase-3 was detected in CQ and CCI-779 treated cells when compared to a staurosporine treated control (Figure S3A). Addition of the broad spectrum caspase inhibitor Q-VD-OPh failed to rescue cell death by the combination of CQ and CCI-779 in a clonogenic assay, suggesting that RCC cells were not dying by apoptosis.

**Figure 3:**
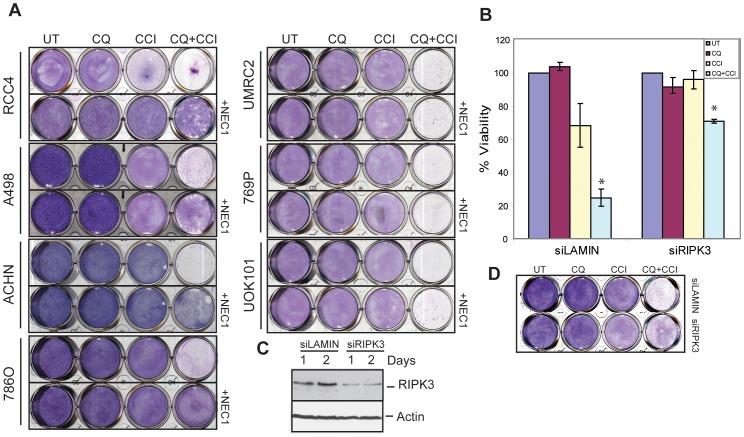
Cell death induced by autophagy and mTOR inhibition is RIPK-dependent necroptosis. (A) Clonogenic survival assay showing Nec1 pretreatment rescues clonogenic survival. RCC cells were pretreated for one hour with Necrostatin1 (Nec1) followed by the addition of CQ, CCI-779 or combination of CQ and CCI-779 to the media. After 18 hours media was changed to normal culture media and cells were allowed to recover for 5 days. In 4 of 7 RCC cell lines (RCC4, A498, ACHN, 786O), Nec1 pretreatment rescued clonogenic survival from CQ and CCI. (B) Viability graph showing that siRNA knockdown of RIPK3 in RCC4 prevented cell death induced by an 18 hour drug treatment of combination of CQ and CCI-779 as compared to siRNA control siLamin (*p = 0.007). Error bars represent ± S.D. (C) Western blots of RNA knockdown of RIPK3. RCC4 cells were transfected with either Lamin- or RIPK3-siRNA and analyzed for levels of RIPK3 over 2 days. (D) Clonogenic survival assay showing Nec1 pretreatment or knockdown of essential necroptosis protein RIPK3 enhanced survival in the combination of CQ and CCI-779 as compared to cells without Nec1 or with siRNA control siLamin.

When examined by electron microscopy, CQ and CCI treated cells displayed signs of plasma membrane rupture indicative of a necrotic phenotype (data not shown). Death may also occur by a programmed form of necrosis, necroptosis, which requires RIPK1 and RIPK3 activated by death receptors [34] or death receptor-independent mechanisms [35]. To assess if CQ and CCI-779 induced necroptosis, human RCCs were treated with CQ and CCI-779 in the absence and presence of the specific RIPK1 and necroptosis inhibitor Necrostatin-1 (Nec1) [36]. Nec1 alone had no effect, but dramatically restored clonogenic survival by the combination of CQ and CCI-779 in 4 of 7 RCC cell lines (Figure 3A). Knockdown of RIPK3 in RCC4 (Figure 3C) also suppressed cell death (Figure 3B and S3C) and enhanced clonogenic survival (Figure 3D), confirming cell death by CQ and CCI-779 was RIPK-dependent necroptosis. RCC4 cells failed to cleave caspase-8 in response to CQ and CCI further supporting necroptotic cell death (Figure S3D). Similarly, RCC4 cells remained viable in response to the combination of TNF-α and Z-VAD-FMK further supporting the conclusion that RCC4 cells are defective of necroptosis induced by TNF-α, (Figure S3E) possibly due to defective TNF-α signaling. This was in stark contrast to control FADD-deficient Jurkat cells, which died in response to TNF-α and Z-VAD-FMK (Figure S3E).

### Mitochondrial ROS production causes necroptosis

**Figure 4:**
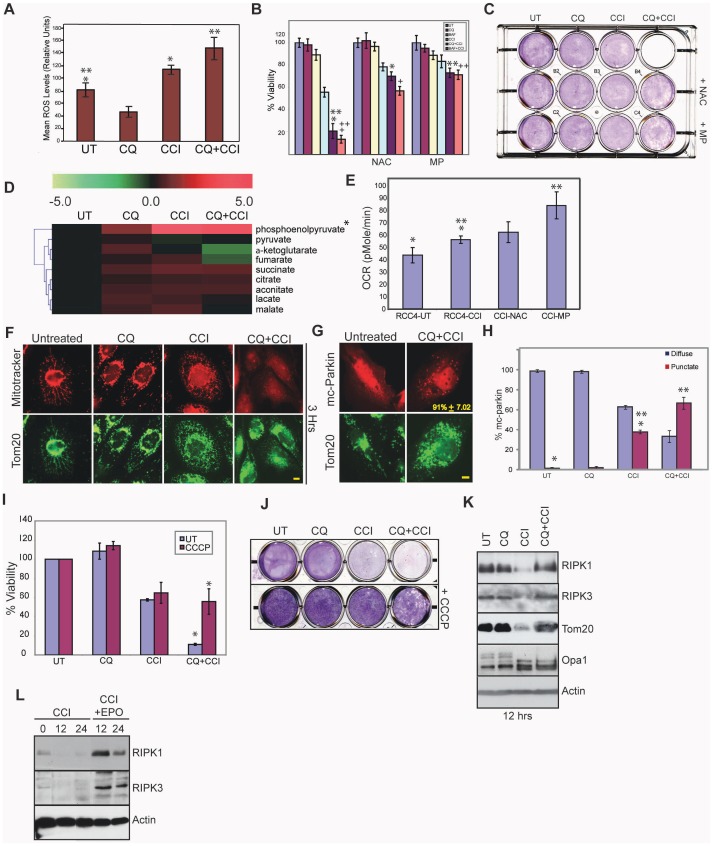
Mitochondrial ROS production causes cell death with coordinate mTOR and autophagy inhibition. (A) Mean ROS levels in RCC4, untreated, CQ, CCI-779, or combination of CQ and CCI-779 after 6 hours by flow-cytometry using the ROS sensor DCF-DA (*p = 0.003,**p = 0.004). (B) Viability graph of RCC4 cells after 18 hours with 40 µM CQ 40 µM CCI-779, 4 nM bafilomycin A1 (BAF) or combination of CQ with CCI-779 or BAF. Cell death is prevented with the addition of 5 mM n-acetyl-cysteine (NAC) or 8 mM methyl-pyruvate (MP) (*p = 0.004,**p = 0.002,+p = 0.0003,++p = 0.0002). (C) Clonogenic survival assay of B. (D) Heat map showing relative pool sizes of TCA cycle intermediates measured by LC-MS after 3 hour drug incubation. RCC4 cells with CCI-779 and CQ plus CCI-779 showed accumulation of phosphoenolpyruvate (PEP) and a depletion of pyruvate (*p = <0.05). (E) Oxygen consumption rate (OCR) of RCC4 cells with DMEM control, 40 µM CCI-779, CCI-779+5 mM NAC or CCI-779+8 mM MP after a 3 hour drug incubation (*p = 0.002,**p = 0.012). (F) RCC4 cells were stained with the mitochondrial potential-dependent dye Mitotracker Red and Tom20 after 3 hours of drug incubation. Scale bar = 10 microns. (G) RCC4 cells expressing m-cherry-Parkin. Levels of mitophagy were quantitated after a 9 hour drug treatment by counting the percent of cells with punctuate m-cherry-Parkin expression. Scale bar = 10 microns. % colocalization of mc-Parkin and Tom 20 +/− S.D. shown as yellow numbers. (H) CCI-779 induces mitophagy (38%,*p = 9.52×10^−6^). Levels of mitophagy are enhanced in the combination of CQ and CCI-779 (60%,**3.752×10^−5^). (I) RCC4 cells pretreated for one hour with 10 µM CCCP followed by addition of CQ, CCI-779 or combination of CQ and CCI-779 to the media with and without CCCP (*p = 0.002). (J) CCCP enhanced clonogenic survival of cells treated with the combination of CQ and CCI-779 for 18 hours. (K) Western blots showing loss of RIPK1, RIPK3 and the mitochondrial marker Tom20 with CCI-779 at 12 hours, which is blocked by combination of CQ and CCI-779 as well as cleavage of the mitochondrial marker Opa1. (L) Western blot showing RIPK1 and RIPK3 degradation is prevented by 100 nm epoxomicin.

Necroptosis is often associated with mitochondrial ROS production [34], [37], [38] suggesting that combining CQ and CCI-779 may cause lethal mismanagement of oxidative stress. CCI-779 alone induced ROS as indicated by the ROS sensor DCF-DA (Figure 4A) and by increased MitoSOX™ Red superoxide detector staining of mitochondria (Figure S4A). ROS production was elevated when CCI-779 was combined with CQ (Figure 4A and S4A). To determine if ROS production by CCI-779 and CQ was required for cell death, we tested for rescue of cell death by the ROS scavenger, N-Acetyl Cysteine (NAC). NAC, but not ROS scavenger-inactive N-Acetyl Alanine (NAA), inhibited necroptosis and enabled clonogenic survival to the combination of CCI-779 and CQ or Bafilomycin A1 (BAF) that prevents maturation of autophagic vacuoles [39] (Figure 4B–C and S4B–F). Since mTOR inhibition suppresses glycolysis [40], [41] and presumably production of the mitochondrial substrate pyruvate, we tested if supplementation with methyl-pyruvate (MP) prevented cell death. Indeed, MP rescued cell death and clonogenic survival with CCI-779 and CQ or BAF (Figure 4B–C and S4B–C), suggesting that mitochondria are substrate-limited and that supplementing mitochondrial substrates supports normal respiration and survival.

### mTOR inhibition elevates oxidative metabolism and demand for substrates

To examine the impact of CCI-779 and glycolysis inhibition on cellular metabolism, relative pool sizes of glycolytic and tricarboxylic acid (TCA) cycle intermediates were analyzed by LC-MS. Pool size measurements showed accumulation of phosphoenolpyruvate (PEP) and depletion of pyruvate and other TCA cycle metabolites in RCC4 cells with CCI-779 or the combination of CQ and CCI-779 (Figure 4D). This suggests that CCI-779 inhibits glycolysis upstream of pyruvate.

As glycolysis inhibition can cause compensatory upregulation of mitochondrial respiration leading to increased ROS production [42], we examined if mTOR inhibition increased mitochondrial function, and thus mitochondrial oxidative phosphorylation by measuring the oxygen consumption rate (OCR), which is a direct readout of mitochondrial function. OCR was low in RCC4 cells, consistent with high glycolytic metabolism, and the addition of CCI-779 increased OCR, suggesting that blocking glycolysis through mTOR inhibition switched cellular metabolism more toward oxidative phosphorylation (Figure 4E). This suggested that CCI-779 and glycolysis inhibition caused a compensatory upregulation of mitochondrial respiration, possibly as an alternative ATP source, which elevated mitochondrial substrate demand. This increase in OCR was augmented by addition of MP, while the ROS scavenger NAC had no effect (Figure 4E), indicating that MP directly supported mitochondrial respiration and survival primarily by overcoming TCA substrate depletion.

### mTOR inhibition induces mitophagy that is blocked by CQ

Combination of CQ and CCI-779 induced cell death that was reversed by inhibition of RIPKs, NAC or MP, suggesting that it resulted from mismanagement of mitochondrial metabolic function. To investigate this, mitochondrial morphology was examined by electron microscopy. Mitochondria appeared normal with CQ alone, and CCI-779 caused mitochondrial fragmentation that persisted in combination with CQ (Figure S5A). Fragmented Mitochondrial were not sequestered in lysosomes or autophagosomes implying that they might be the source of ROS production. To assess if this was associated with mitochondrial dysfunction, mitochondrial membrane potential was measured with the potential dependent dye Mitotracker Red in comparison to the mitochondrial marker Tom20. CCI-779, and to a much greater extent CQ and CCI-779, disrupted membrane potential and fragmented mitochondria indicating mitochondrial dysfunction (Figure 4F).

Depolarized, fragmented mitochondria are degraded by mitophagy. To determine if mTOR inhibition induced mitophagy, RCC4 cells were transfected with the mitophagy marker mCherry-Parkin, which translocates to depolarized mitochondria and serves as a signal for mitochondrial degradation via autophagy [43]. CCI-779 induced Parkin translocation that was increased by the combination of CQ and CCI-779 (Figure 4G–H). This suggests that CQ blocked mitophagy, preventing clearance of ROS-producing mitochondria resulting from mTOR inhibition.

### Mitochondria are required for necroptosis

As CCI-799 promotes mitochondrial elimination by mitophagy, and blocking mitophagy with CQ leads to mitochondrial ROS production and necroptosis, we examined whether blocking mitochondrial respiration and promoting mitophagy before drug treatment would prevent necroptosis. Uncoupling mitochondria with carbonyl cyanide m-chlorophenylhydrazone (CCCP) causes depolarization, Parkin translocation and elimination of most but not all cellular mitochondria by mitophagy (Figure S5C–D) [44], [45]. Pretreatment of RCC4 cells with CCCP prevented necroptosis by CQ and CCI-779 and restored clonogenic survival (Figure 4I–J and S5B). CCI-779 caused a profound reduction of RIPK1, RIPK3 and the mitochondrial marker Tom20 which was prevented by CQ (Figure 4I). Loss of RIPK1 and RIPK3 was blocked by the proteasome inhibitor epoxomicin indicating degradation by the ubiquitin proteasome system. CCI-779 also caused cleavage of the inner mitochondrial protein Opa1, indicative of mitochondrial fragmentation (Figure 4K). This suggested that elimination of RIPKs through both the ubiquitin proteasome system and elimination of mitochondria by mitophagy is a resistance mechanism to mTOR inhibition that prevents ROS- and RIPK-mediated necroptosis, which is reversed by autophagy/mitophagy inhibition with CQ.

### mTOR inhibition blocks Nrf2 nuclear translocation and antioxidant defense

**Figure 5:**
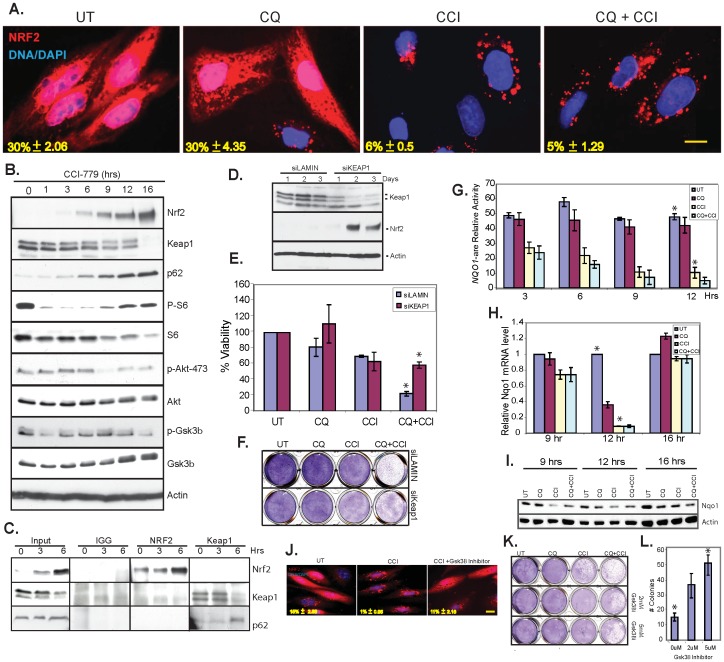
mTOR inhibition blocked Nrf2 nuclear translocation and anti-oxidant defense creating dependency on autophagy for survival. (A) Ectopic expression of Nrf2 (red) and nuclear staining with Dapi (blue) after a 6 hour drug incubation. Yellow numbers represent the percent of cells with nuclear Nrf2 +/− the standard deviation. Scale bar = 10 µM. (B) Western blot time course of CCI-779 treated RCC4 cells. Nrf2 and p62 were induced with CCI-779 treatment, while Keap1 levels go down. S6 lost phosphorylation after 1 hour of CCI-779 treatment, while total S6 remained the same. Akt lost phosphorylation at serine 473 after 9 hours of CCI-779 treatment. Gsk3β lost its phosphorylation after 1 hour of CCI-779 incubation. (C) Immunoprecipitation of IGG control antibody, Nrf2, and Keap1 after 0, 3, 6 hours CCI-779 incubation. Nrf2 and Keap1 did not co-immunoprecipitate while p62 co-immunoprecipitates with Keap1. (D) Western blot of siRNA knockdown of Keap1. RCC4 cells were transfected with either Lamin or Keap1 siRNA and analyzed for levels of Keap1. Knockdown of Keap1 induced Nrf2. (E) Viability graph showing knockdown of Keap1 rescued cell death induced by CQ and CCI-779 combination as compared to the Lamin control after 18 hours. (*p = 0.003) (F) Clonogenic survival assay of RCC4 cells with RNA knockdown of Keap1 or Lamin control. (G) CCI-779 regulated transcriptional activity of Nrf2. RCC4 cells were transfected with the NQO1-ARE promoter firefly luciferase and *Renilla* luciferase as a control. Relative NQO1 mRNA level was measured. (*p = 2.353×10^−5^) (H) CCI-779 regulated mRNA level and protein level (I) of the Nrf2 target gene *Nqo1*. (Western blot of Nqo1). (*p = 0.003). (J) Localization of ectopically expressed Nrf2-RFP. CCI-779 caused exclusion Nrf2 from the nucleus. Pretreatment with a Gsk3β inhibitor restored nuclear localization of Nrf2. Yellow numbers represent % nuclear localization +/− S.D. (K) Clonogenic survival assay showing enhanced survival of CQ and CCI-779 treated cells when treated in combination with either 2 or 5 µM Gsk3β inhibitor. (L) Quantitation of colonies formed after the combination of CQ and CCI-779 with the Gsk3β inhibitor (*p = 1.01×10^−4^).

If mitochondrial ROS production and RIPK activation is the mechanism by which CQ and CCI-779 promote necroptosis, we questioned why this was not prevented by antioxidant defense pathways. Nrf2 is a critical transcription factor controlling expression of antioxidant defense genes [46]. Nrf2 binds Keap1, a member of the Keap1-Cullin3-E3 ubiquitin ligase complex that targets Nrf2 for degradation in proteasomes [47]. Nrf2 is activated by the adaptor protein p62 that binds and inhibits Keap1, releasing and stabilizing Nrf2 causing its nuclear translocation and antioxidant response [48], [49]. In RCC4 cells, ectopically expressed RFP-Nrf2 was present in the nucleus and cytoplasm of approximately one third of RCC4 cells under normal growth conditions, suggesting that Nrf2 might be constitutively active, which was not altered by CQ (Figure 5A). This is consistent with expression of endogenous p62 that is bound to Keap1 (Figure 5C). CCI-779 alone and in combination with CQ suppressed nuclear translocation of RFP-Nrf2 (Figure 5A) and endogenous Nrf2 (Figure S6). CCI-779 upregulated Nrf2 and p62 and dowregulated Keap1 (Figure 5B), suggesting that CCI-779 relieved Nrf2 from Keap1 by upregulating p62, but also blocked Nrf2 nuclear translocation and activation of the antioxidant response. Endogenous p62 and Keap1 co-immunoprecipitated when mTOR was inhibited, whereas Nrf2 and Keap1 did not (Figure 5C). Knockdown of Keap1 induced Nrf2 accumulation and rescued cell viability and clonogenic survival to subsequent CCI-779 and CQ treatment (Figure 5D–F). This suggests that Nrf2 was active in RCC4 cells due to p62 inactivation of Keap1, but that mTOR inhibition prevents Nrf2 nuclear translocation and antioxidant defense, creating the need for autophagy to mitigate ROS.

To determine the transcriptional activity of Nrf2, a firefly luciferase reporter gene activity assay for the Nrf2 target gene *Nqo1* was performed. Under normal growth conditions, *Nqo1* activity was high and this was repressed by CCI-779 and CCI-779 plus CQ (Figure 5G). Similarly, *Nqo1* mRNA (Figure 5H) and protein levels (Figure 5I) were decreased by CCI-779 (12 hours). Levels of *Nqo1*1 mRNA went back up at 16 hours, presumably due to high levels of ROS production at this time, Nrf2-independent activation or activation of the mTOR negative feedback loop as evident by phosphorylation of S6 (Figure 5B).

Inhibition of Nrf2 nuclear localization is caused by Gsk3β phosphorylation [50], and CCI-779 suppressed phosphorylation of Gsk3β on serine 9, indicative of activation (Figure 5B). Pretreatment with a Gsk3β inhibitor partially rescued the block to Nrf2 nuclear localization caused by CCI-779 (Figure 5J) and clonogenic survival to CCI-779 and CQ (Figure 5K–L). Thus, mTOR inhibition suppresses antioxidant defense, which is compensated for by elimination of RIPKs and induction of autophagy. CQ blocks autophagy and RIPK degradation, causing the persistence of ROS-producing mitochondria, RIPK activation, and necroptosis.

## Discussion

RCC is a disease with high mortality and allosteric mTOR inhibitors have efficacy but are not curative [22], [23]. Strategies being implemented to improve outcome are combining rapalogs with inhibitors targeting other pathways activated in RCC, such as the VEGF pathway [51] or mTOR negative feedback [52]. Alternatively, if autophagy activation by mTOR inhibitors promotes tumor cell survival, then therapeutic strategies to block autophagy downstream of mTOR are warranted [15], [16].

To establish if autophagy suppression improves anti-cancer activity of mTOR inhibitors, we determined the functional role of autophagy activation by rapalogs and that autophagy inhibition enhanced the anti-tumor activity. Genetic or pharmacologic autophagy suppression increased the cytotoxicity of CCI-779, supporting a pro-survival role for autophagy. These findings demonstrate the utility of coordinate mTOR and autophagy inhibition.

It has become apparent that many aggressive cancers have upregulated autophagy and are dependent on autophagy for survival [11]. Autophagy in Ras-driven cancers and BCR-Abl-driven leukemias is required for survival [4], [26], [29], [33]. Although the mechanism of autophagy addiction is not yet known, evidence suggests that autophagy supplies mitochondrial substrates to sustain the TCA cycle and respiration, without which mitochondria malfunction, causing an energy crisis and cell death [4], [29]. Although human RCC does not typically have Ras mutations, loss of PTEN activates Ras effector pathways. Consistent with autophagy addiction, RCC cell lines have high basal autophagy and are dependent on autophagy for growth.

CQ and HCQ interfere with lysosome function, blocking the degradation of the products of autophagy [53]. Although CQ can have other activities, it is clear that CQ blocks autophagic flux and enhances cytotoxicity of CCI-779, and that knockdown of Atg7 acted similarly. This suggests that inhibition of autophagy is effective at sensitizing RCC cells to mTOR inhibition. CQ or genetic inhibition of autophagy also sensitizes cancer cells to cancer therapeutics, although the mechanisms are not known. These include the proteasome inhibitor bortezomib (Velcade) [6], [10], [54], alkylating agents [24], imantinib (Gleevec) [55], mTOR inhibitors [30] and AKT inhibitors [28], [56]. In contrast, in some settings excessive autophagy is associated with cell death, and small molecules have been identified that promote this form of cell death specifically in VHL-negative RCC cell lines [57], [58]. It will be of interest to establish the mechanism by which excessive autophagy stimulation may lead to cell death as well as to exploit the utility of autophagy inhibitors to promote cell death.

In most cases, including mammalian development, autophagy promotes survival [1]. Rare circumstances where autophagy inhibition promotes survival may be due to upregulation of p62 and activation of Nrf2 [11]. Autophagy can prevent apoptosis, necrosis [14], [59], and now also necroptosis. Necroptosis is a specialized form of programmed necrosis that must be inactivated to prevent embryonic lethality [60], [61], [62]. Necroptosis is also part of host defense against virus infection [63], [64], [65]. We have delineated here that necroptosis is induced by mTOR inhibition, mitochondrial respiration and RIPKs, when antioxidant defense by Nrf2 and mitophagy are blocked. How RIPKs are activated is not clear, but they may be sensors for mitochondrial distress such as substrate limitation either upstream or downstream of ROS. Autophagy-supplied mitochondrial substrates may preserve normal mitochondrial function while eliminating RIPKs through the ubiquitin proteasome system, and distressed mitochondria through mitophagy, enabling survival to mTOR inhibition. Independent of the mechanism of RIPK activation, these findings demonstrate that necroptosis is relevant for cancer therapy, as emerging evidence suggests [66], [67]. It will be important to establish if necroptosis can be exploited, as the apoptosis pathway has been [68], for the development of targeted cancer therapeutics.

**Figure 6:**
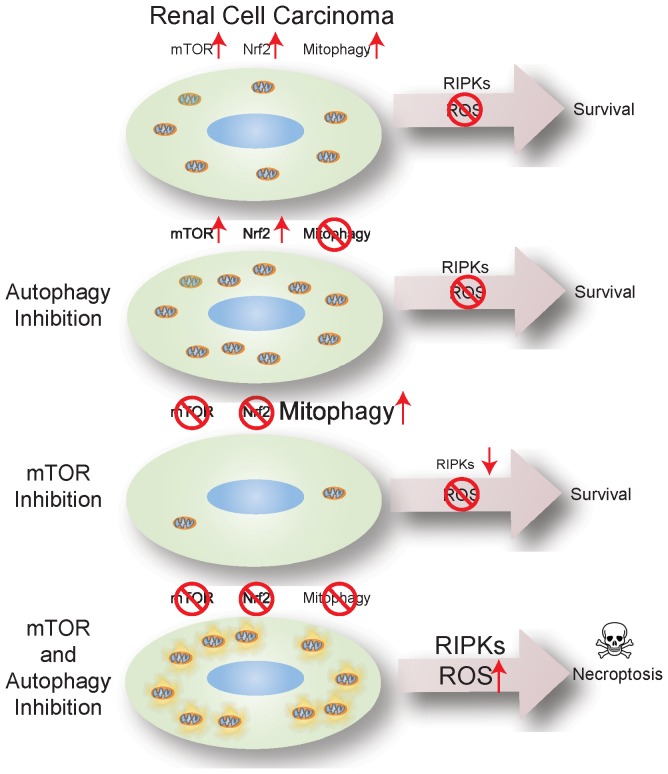
Model depicting mechanism of necroptosis induction by mTOR and autophagy inhibition. Renal carcinoma cells have activated mTOR, Nrf2 antioxidant defense and autophagy under basal conditions sufficient to manage ROS and survive. Autophagy inhibition with CQ blocks clearance of damaged mitochondrial through mitophagy. ROS is mitigated through the Nrf2 antioxidant defense pathway. mTOR inhibition promotes respiration and inhibits Nrf2 antioxidant defense. RIPKs are degraded by the ubiquitin proteasome system and damaged mitochondrial are cleared by mitophagy to maintain ROS homeostasis and cell survival. Pharmacological inhibition of both autophagy with CQ and mTOR with CCI-779 causes loss of antioxidant defense via Nrf2 and persistence of RIPKs and mitochondria leading to RIPK activation and ROS-mediated cell death by necroptosis.

A key determinant of mTOR inhibition-triggered necroptosis was the blockade of Nrf2 nuclear translocation and antioxidant defense. There were no deleterious consequences to mTOR inhibition alone and blockade of Nrf2 because of elimination of RIPKs and stimulation of mitophagy to promote elimination of ROS-producing mitochondria (Figure 6). CQ alone prevents mitophagy, but active Nrf2 was sufficient to manage the additional ROS load. With mitophagy blocked, the resulting persistence of ROS-producing mitochondria and RIPKs was lethal when mTOR inhibition prevented Nrf2 activation (Figure 6). This critical balance between Nrf2 antioxidant defense and mitophagy to manage ROS and RIPKs is uncoupled by combining CQ with mTOR inhibition (Figure 6). In RCC, p62 is expressed and bound to Keap1 enabling Nrf2 activation. Mutations activating Nrf2 or inactivating the inhibitor of Nrf2, Keap1, are found in human cancers [46], [69]. Thus, p62 expression similar to Nrf2 activation or Keap1 inactivation may enhance antioxidant defense and cancer cell survival. It is intriguing to speculate that cancers with Nrf2 pathway activation may be vulnerable to coordinate mTOR and autophagy inhibition and RIPK- and ROS-mediated necroptosis, and that inactivation of RIPKs and necroptosis is a mechanism of resistance to cancer therapy.

## Materials and Methods

### Ethics statement

Tumor assays in mice were performed using Institutional Animal Care and Use approved protocols and was approved by IACUC committee members at the University of Medicine and Dentistry of New Jersey (UMDNJ). Tumor assays performed in this manuscript were approved under the protocol, I10-064-8, “Use of Mice for Tumorigenicity.” Mice were monitored for overall health in response to drug treatment and sacrificed when tumor size exceeded 400 mm^3^. All efforts were made to minimize suffering during the experiment. Full details and experimental conditions are listed below.

### Cell lines and culture conditions

RCC4, UMRC2, UOK101, ACHN, 786O and A498 were cultured in DMEM without sodium pyruvate (Invitrogen), 10% FBS, and 1% Penn/Strep at 37°C and 5% CO_2_. 769P was cultured in RPMI media (Invitrogen) with 10% FBS, 1% Penn/Strep at 37°C and 5% CO_2_. All RCC cell lines were obtained from Dr. William Kaelin. All RCC cell lines were originally purchased from the ATCC except UOK101 [70] and UMRC2 [71]. I2.1 cells were cultured in RPMI media with 10% FBS, 1% Penn/Strep and were purchased from the ATCC. For viability assays, cells were collected and counted by trypan blue exclusion. CQ and CCI-779 were used at 40 µM for all in vitro experiments unless otherwise noted. For clonogenic survival assays, cells were subjected to 18 hour drug treatments (unless otherwise stated) and then allowed to recover in normal media for 5 days. Colonies were fixed in methanol, stained with Geimsa (Sigma), and photographed.

### Drug administration in mouse allografts

Tumor assays were performed using Institutional Animal Care and Use approved protocols. RCC4 cells (10^7^ cells/injection) were inoculated subcutaneously to form tumors. Mice with tumors (150 mm^3^) assorted to four groups (5 mice/group). Vehicle or CQ (65 mg/kg/d, 7 d/week, 3 cycles) or CCI-779 (20 mg/kg/d, 5 d/week, 3 cycles) alone or in combination was administrated intraperitoneally. CCI-779 was prepared in the provided diluent and further diluted in PBS. CQ was prepared in PBS.

### Western blotting, immunofluorescence, and electron microscopy

Western blotting and immunofluorescence were performed as previously described [72]. For fluorescent visualization of mCherry-Parkin localization, cells were plated on cover-slips, subjected to drug incubation and fixed in 4% paraformaldehyde (mCherry-Parkin) for 15 min at room temperature. For fluorescent visualization of RFP-NRF2, cells were plated on cover-slips, subjected to drug incubation and fixed in methanol for 10 minutes at −20°C. For LC3 and Nrf2 immunostaining cells with fixed in methanol for 20 min at −20°C and stained with primary antibody (LC3 antibody, Novus Biologicals, NB600-1384, 1∶2000) or NRF2, Santa Cruz, sc.13032 1∶250) for 2 hours at 37°C followed by FITC or TRITC tagged anti-rabbit (Jackson Immuno Research, 1∶50 dilution). For mitochondrial imaging by EM, cells were fixed in 0.1 M cacodylate buffer with 2.5% glutaraldehyde, 4% paraformaldehyde, and 8 µM CaCl_2_ and analyzed by a JOEL 1200EX electron microscope as described previously [14].

### Autophagy and mitophagy assessment

For quantification of autophagy levels, cells were immunostained with LC3 antibody as described above. Autophagy levels were quantitated by scoring a minimum of 200 cells in three replicate experiments. A cell with more than 20 LC3 puncta was defined as positive for autophagy. Images were analyzed at 1000× magnification. The percentage of cells with mCherry-Parkin translocation was quantified by determining the number of cells displaying punctate fluorescence out of a population of 200 fluorescent cells replicated in three independent experiments.

### ROS assays

ROS levels were determined by washing proliferating cells once with Hanks balanced salt solution (HBSS) (Invitrogen) and incubating in 10 µM 2′-7′-dichlorodihydrofluorescene diacetate (DCF-DA, Molecular Probes, Invitrogen) in pre-warmed HBSS for 15 min at 37°C followed by measurement of mean fluorescence by flow-cytometry (FC500; Beckman Coulter). Three independent experiments were performed analyzing 10,000 cells (per genotype) in each experiment. For visualization of ROS by fluorescence, cells were plated on coverslips and subjected to drug incubation. Following drug incubation 5 µM MitoSOX™ Red superoxide indicator (Molecular Probes, Invitrogen) was added to the media and incubated 10 minutes. Cells were washed with PBS and imaged at 600× magnification.

### RNA interference

RCC4 cells were transfected with siGENOME SMARTpool siRNA targeted against human RIPK3 (NM_006871), Keap1 (NM_012289) or Lamin A/C control siRNA (Dharmacon Research, Chicago, IL; M-047628-00-0010) using lipofectomine transfection reagent (Invitrogen). 24, 48 or 72 hours post-transfection, cells were incubated with drugs and viability was assessed by trypan blue (Invitrogen) exclusion or by clonogenic assay. Total protein was isolated in parallel and analyzed by Western blotting to assess knock-down of target genes.

For shRNA knockdown of Atg7, pLKO.1-derived vectors with shRNAs targeting Atg7 were purchased from Sigma-Aldrich (TRCN0000007584, and TRCN0000007587). Control shRNAs against RFP and Luciferase were generously provided by the Broad Institute. Virus was produced using a second-generation packaging system in 293T cells as described [73].

### Luciferase reporter gene assay

Luciferase reporter assays were performed as described previously [49]. Briefly, RCC4 cells were transiently transfected with 1.6 µg of the Nqo1 ARE luciferase and 0.01 µg *Renilla* luciferase control (hRluc/TK) plasmids. Approximately 24 hours post transfection, cells were treated with CQ, CCI-779 or combination of CQ and CCI-779 for 12 hours. Relative luciferase activities were calculated using Promega Dual Luciferase kit (Promega Corporation, WI) and a TD 20/20 luminometer (Turner Designs, Sunnyvale, CA) and normalized to *Renilla* luciferase values.

### mRNA extraction and real-time qRT-PCR

Total mRNA was extracted using TRIzol (Invitrogen) according to the manufacturer's instructions. Using equal amounts of mRNA and the Transcriptor First Strand cDNA synthesis kit (Roche), cDNA was generated and used for real-time quantitative reverse transcription-PCR (qRT-PCR). The TaqMan probes were obtained from the universal probe library (Roche): hNQO1, no. 87; and hGAPDH (glyceraldehyde-3-phosphate dehydrogenase), no. 25. Forward and reverse primers for hNQO1, and hGAPDH were synthesized by Integrated DNA Technologies: sequences are as follows: hNQO1, ATGTATGACAAAGGACCCTTCC (forward) and TCCCTTGCAGAGAGTACATGG (reverse); and hGAPDH, CTGACTTCAACAGCGACACC (forward) and TGCTGTAGCCAAATTCGTTGT (reverse). Real-time PCR was performed as followed: one cycle of predenaturation (95°C for 5 min), 45 cycles of amplification (95°C for 10 s and 60°C for 20 s), and a cooling program of 50°C for 30 s. Reactions were done in triplicate, and the experiment was repeated three times. The data were expressed as relative mRNA levels and normalized to GAPDH.

### Mitochondrial membrane potential assay

RCC4 cells were seeded at 1.5×10^5^ cells per cell in a 12-well plate. Cells were treated with CQ, CCI-779 or combination for 3 hours. Following drug treatment, cells were stained with 50 nM Mitotracker Red (Molecular Probes/Invitrogen) for 20 minutes at 37°C in DMEM with 10% FBS and 1%P/S. Cells were washed for 15 minutes in DMEM and visualized (data not shown) or fixed in 4% PFA for 15 minutes at room temperature. Cells were then stained with Tom20 as described above.

### OCR rate measurement

OCR rates were measured using Seahorse Biosciences extracellular flux analyzer (XF24-3). Cells were seeded at 7.5×10^4^ cells per well (0.32 cm^2^) in XF24 plates in 250 µL of DMEM (10% FBS, 1% Pen-Strep) and incubated for 20–24 h at 38.5°C and 8.5% CO_2_ prior to XF assay and OCR measurements were made as described previously [42].

### Metabolomic analysis by LC-MS

Pool size measurements in major TCA cycle intermediates by LC-MS were performed as described previously [74]. Briefly, cells were cultured in complete media (DMEM, 10% dialyzed FBS, 1% Pen-Strep) for 20–24 h, after which media was replaced with DMEM containing drugs. Metabolism was quenched by removing the media and adding methanol∶water (80∶20) at −80°C. Extracted metabolites were dried under nitrogen flow, reconstituted in water, and measured by LC-MS. Metabolites were identified by accurate mass (<5 ppm deviation, Exactive) or characteristic fragmentation product (triple quads), in combination with retention time matched to validated standards, using in-house software. Peak intensity data was further analyzed using Excel and MatLab. Metabolite ion count in each replicate sample was normalized to the cell number and the normalized ion counts were expressed as fold change normalized to the untreated control. To find statistical significance fold change ratios were log_2_ transformed, and p-value calculated using two-tailed distribution. A p-value<0.05 was considered statistically significant. Major TCA metabolites were selected and cell number-normalized pool sizes were calculated as an average of three replicate measurements.

### Antibodies and chemicals

The following antibodies were used: Actin, RIPK3, Atg7 (Sigma); LC3 (Novus), p62 (Biomol and a rabbit polyclonal antibody [4]); active caspase-3 (Asp175), S6, phospho-S6 (ser 235/236), AKT, phospho-AKT 473, phospho-GSK3β (ser 9), GSK3β (Cell Signaling), Nrf2 (H300), Keap1 (E20), Tom20 (F10) and Nqo1 (Santa Cruz), RIPK1, Opa1 (BD Transduction). The following chemicals were obtained as follows: CCI-779 (Wyeth and Sigma), chloroquine disulfate salt (CQ), bafilomycin A1, Necrostatin-1, N-acetyl-cysteine, N-acetyl-alanine, CCCP, and methyl-pyruvate (Sigma), Mitotracker Red FM (Invitrogen), Torin1 (generously provided by Nathanael S. Gray), and the Gsk3β Inhibitor XII (TWS119) (EMD Chemicals).

### Statistics

Mean ± standard deviation (s.d.) was presented, except in Figure 2D, where mean ± standard error was presented. Data shown are the results of three independent experiments. P values were calculated using a paired two-tailed students t-test. P<0.05 was considered significant.

## Supporting Information

Figure S1 **Renal carcinoma cell lines have high basal autophagy and are sensitive to the combination of CQ and CCI-779.** (Related to Figure 1) (A) Endogenous LC3 immunostaining in human RCC cell lines (hRCCs). hRCCs were left untreated or treated with 40 µM CQ for 3 hours. Levels of autophagy were quantitated by counting the % of cells with more than 20 LC3 puncta as positive. All hRCCs have high basal autophagy and show 100% punctation with treated with CQ indicating high autophagic flux. Scale bar = 10 µm. (B) Representative stills from Movies S1, S2, S3, S4, S5, S6, S7, S8, S9, S10, S11, S12, S13, S14, S15, S16. RCC4 and A498, with and without VHL, were incubated with 40 µM CCI-779, 40 µM CQ or combination of CQ and CCI-779 for 18 hours. Combination of CQ and CCI-779 dramatically induced cell death in RCC cells.
(TIF)

Figure S2 **Autophagy and mTOR inhibition induces cell death. (Related to** **Figure 2** **)** (A) Representative images of Fig. 2A and B after 7 days of drug incubation demonstrating toxicity of 20 µM CQ and 20 µM CCI-779 or 20 µM CQ and 250 nM Torin1. Scale bar = 50 microns. (B) Representative images of Fig. 2D showing RCC4 cells with control knockdown (shRfp, shLuc) or knockdown of Atg7 (shAtg7#1,2). Atg7 knockdown sensitized RCC4 to 40 µM CCI-779. Scale bar = 50 microns.
(TIF)

Figure S3 **Autophagy and mTOR inhibition induces necroptosis. (Related to** **Figure 3** **)** (A) Western blot for cleaved caspase-3 in RCC4 treated with CQ, CCI-779 and combination of 40 µM CQ and 40 µM CCI-779 at 16 hrs. As a control RCC4 cells were treated with 4 µM staurosporine for 10 hours. (B) Clonogenic survival assay showing that the pan-caspase inhibitor Q-VD-OPh does not rescue cell death induced by CQ and CCI. RCC4 was pretreated with 20 µM Q-VD-OPh for 1 hour followed by the addition of 40 µM CQ and 40 µM CCI to the media. After an 18 hour drug treatment media was changed to normal growth media and cells were allowed to recover for 5 days. (C) Representative images of Fig. 3B. Knockdown of RIP3K in RCC4 rescues cell death induced by 40 µM CQ and 40 µM CCI as compared to Lamin control knockdown. Scale bar = 50 µM. (D) Western blot showing that RCC4 cells do not cleave Caspase-8 in response to 40 µM CQ and 40 µM CCI in comparison to control treated with 100 nM TNF-α and 10 µg/ml cycloheximide. (E) RCC4 and I2.1 (Fadd^−/−^ Jurkat cells) were treated with 100 nM TNF-α and 50 µM Z-VAD-FMK. TNF-α alone induced necroptosis in I2.1 cells. RCC4 remained viable even in the presence of Z-VAD-FMK.
(TIF)

Figure S4 **Mitochondrial ROS production causes cell death that is rescued by NAC and methyl-pyruvate. (Related to** **Figure 4** **)** (A) MitoSox Red superoxide indicator staining of RCC4 cells. 40 µM CCI-779 and combination of 40 µM CQ and 40 µM CCI-779 had high levels of ROS as indicated by MitoSox Red fluorescence after a 6 hour drug incubation. Scale bar = 10 µM. (B) Representative images of Fig. 4B. RCC4 was treated with 4 nM Bafilomycin A1, 40 µM CQ, 40 µM CCI-779 or combination of Bafilomycin A1 and CCI-779 or CQ and CCI-779. The combination of Bafilomycin A1 (BAF) or CQ with CCI-779 induces cell death that is rescue with 5 mM n-acetyl-cysteine (NAC) or 8 mM methyl-pyruvate (MP). Scale bar = 50 µM. (C) Clonogenic survival assay. Combination of BAF and CCI-779 induces cell death that is rescued by NAC and MP. (D) Cell death induced by combination of CQ and CCI-779 is rescued by NAC but not n-acetyl-alanine (NAA). (*p = 2.01×10^−4^) (E) Clonogenic assay of treatment shown in D. (F). Representative photos of D. Scale Bar = 50 µM.
(TIF)

Figure S5 **Mitochondrial are required for necroptosis. (Related to** **Figure 4** **)** (**A**) Electron micrographs of RCC4 treated with 40 µM CQ, 40 µM CCI-779, or combination of CQ and CCI-779. Mitochondrial appear fragmented with CCI-779 or combination of CQ and CCI-779. (**B**) Representative images of Fig. 4I. 10 µM CCCP rescued cell death induced by the combination of CQ and CCI-779 in RCC4 cells. Scale Bar = 50 µM. (**C**) Mitochondrial potential assay showing loss of mitotracker red and membrane potential as compared to total mitochondria (Tom20) after a 6 hour incubation with 10 µM CCCP. Scale Bar = 10 µM. (**D**) CCCP caused m-cherry-Parkin to translocate to the mitochondria. Scale Bar = 10 µM.
(TIF)

Figure S6 **CCI-779 blocks nuclear translocation if Nrf2.** **(Related to** **Figure 5** **)** Endogenous staining of Nrf2 in RCC4 cells after a 6 hour drug incubation. Nrf2 is predominately nuclear when left untreated or with 40 µM CQ. Nrf2 is excluded from the nuclease with 40 µM CCI-779 or combination of CQ and CCI-779. Scale bar = 10 µM.
(TIF)

Movie S1 **CQ synergizes with CCI-779 to promote cell death in human RCC cell lines.** Time-lapse microscopy of human RCC4 (with VHL expression) cell line under normal growth conditions, related to Fig. S1B.
(AVI)

Movie S2 **CQ synergizes with CCI-779 to promote cell death in human RCC cell lines.** Time-lapse microscopy of human RCC4 (with VHL) cell line with 40 µM CQ, related to Fig. S1B.
(AVI)

Movie S3 **CQ synergizes with CCI-779 to promote cell death in human RCC cell lines.** Time-lapse microscopy of human RCC4 (with VHL) cell line with 40 µM CCI-779, related to Fig. S1B.
(AVI)

Movie S4 **CQ synergizes with CCI-779 to promote cell death in human RCC cell lines.** Time-lapse microscopy of human RCC4 (with VHL) cell line with combination of 40 µM CQ and 40 µM CCI-779, related to Fig. S1B.
(AVI)

Movie S5 **CQ synergizes with CCI-779 to promote cell death in human RCC cell lines.** Time-lapse microscopy of human RCC4 (without VHL) cell line under normal growth conditions, related to Fig. S1B.
(AVI)

Movie S6 **CQ synergizes with CCI-779 to promote cell death in human RCC cell lines.** Time-lapse microscopy of human RCC4 (without VHL) cell line with 40 µM CQ, related to Fig. S1B.
(AVI)

Movie S7 **CQ synergizes with CCI-779 to promote cell death in human RCC cell lines.** Time-lapse microscopy of human RCC4 (without VHL) cell with 40 µM CCI-779, related to Fig. S1B.
(AVI)

Movie S8 **CQ synergizes with CCI-779 to promote cell death in human RCC cell lines.** Time-lapse microscopy of human RCC4 (without VHL) cell line with combination of 40 µM CQ and 40 µM CCI-779, related to Fig. S1B.
(AVI)

Movie S9 **CQ synergizes with CCI-779 to promote cell death in human RCC cell lines.** Time-lapse microscopy of human A498 (with VHL expression) cell line under normal growth conditions, related to Fig. S1B.
(AVI)

Movie S10 **CQ synergizes with CCI-779 to promote cell death in human RCC cell lines.** Time-lapse microscopy of human A498 (with VHL) cell with 40 µM CQ, related to Fig. S1B.
(AVI)

Movie S11 **CQ synergizes with CCI-779 to promote cell death in human RCC cell lines.** Time-lapse microscopy of human A498 (with VHL) cell with 40 µM CCI-779, related to Fig. S1B.
(AVI)

Movie S12 **CQ synergizes with CCI-779 to promote cell death in human RCC cell lines.** Time-lapse microscopy of human A498 (with VHL) cell line with combination of 40 µM CQ and 40 µM CCI-779, related to Fig. S1B.
(AVI)

Movie S13 **CQ synergizes with CCI-779 to promote cell death in human RCC cell lines.** Time-lapse microscopy of human A498 (without VHL) cell line under normal growth conditions, Fig. S1B.
(AVI)

Movie S14 **CQ synergizes with CCI-779 to promote cell death in human RCC cell lines.** Time-lapse microscopy of human A498 (without VHL) cell line with 40 µM CQ, related to Fig. S1B.
(AVI)

Movie S15 **CQ synergizes with CCI-779 to promote cell death in human RCC cell lines.** Time-lapse microscopy of human A498 (without VHL) cell with 40 µM CCI-779, related to Fig. S1B.
(AVI)

Movie S16 **CQ synergizes with CCI-779 to promote cell death in human RCC cell lines.** Time-lapse microscopy of human A498 (without VHL) cell line with 40 µM CQ and 40 µM CCI-779 in combination, related to Fig. S1B. Time-lapse microscopy of human RCC4 (Movies S1, S2, S3, S4, S5, S6, S7, S8) and A498 (Movies S9, S10, S11, S12, S13, S14, S15, S16) cell lines under normal growth conditions and in the presence of CQ, the mTOR inhibitor CCI-779, or combination of CQ and CCI-779. Cells were plated on culture dishes and placed in a time-lapse environmental chamber of normal growth conditions. Images were acquired with an Olympus IX71 inverted microscope every 10 minutes for 30 hours. Time-lapse images were converted to movies using ImagePro Plus software (Degenhardt et al., 2006). CQ showed effective synergy with CCI-779 in human RCC cell lines.
(AVI)

## References

[1] Levine B, Kroemer G (2008). Autophagy in the pathogenesis of disease.. Cell.

[2] Tsukamoto S, Kuma A, Murakami M, Kishi C, Yamamoto A (2008). Autophagy is essential for preimplantation development of mouse embryos.. Science.

[3] Kuma A, Hatano M, Matsui M, Yamamoto A, Nakaya H (2004). The role of autophagy during the early neonatal starvation period.. Nature.

[4] Guo JY, Chen HY, Mathew R, Fan J, Strohecker AM (2011). Activated Ras requires autophagy to maintain oxidative metabolism and tumorigenesis.. Genes Dev.

[5] Rabinowitz JD, White E (2010). Autophagy and metabolism.. Science.

[6] Mathew R, Karp CM, Beaudoin B, Vuong N, Chen G (2009). Autophagy suppresses tumorigenesis through elimination of p62.. Cell.

[7] Komatsu M, Waguri S, Koike M, Sou YS, Ueno T (2007). Homeostatic levels of p62 control cytoplasmic inclusion body formation in autophagy-deficient mice.. Cell.

[8] Mathew R, Karantza-Wadsworth V, White E (2007). Role of autophagy in cancer.. Nat Rev Cancer.

[9] Jin S, White E (2008). Tumor suppression by autophagy through the management of metabolic stress.. Autophagy.

[10] Mathew R, White E (2011). Autophagy in tumorigenesis and energy metabolism: friend by day, foe by night.. Curr Opin Genet Dev.

[11] White E (2012). Deconvoluting the context-dependent role for autophagy in cancer.. Nat Rev Cancer.

[12] Mathew R, Kongara S, Beaudoin B, Karp CM, Bray K (2007). Autophagy suppresses tumor progression by limiting chromosomal instability.. Genes Dev.

[13] Karantza-Wadsworth V, Patel S, Kravchuk O, Chen G, Mathew R (2007). Autophagy mitigates metabolic stress and genome damage in mammary tumorigenesis.. Genes Dev.

[14] Degenhardt K, Mathew R, Beaudoin B, Bray K, Anderson D (2006). Autophagy promotes tumor cell survival and restricts necrosis, inflammation, and tumorigenesis.. Cancer Cell.

[15] White E, DiPaola RS (2009). The double-edged sword of autophagy modulation in cancer.. Clin Cancer Res.

[16] Amaravadi RK, Lippincott-Schwartz J, Yin XM, Weiss WA, Takebe N (2011). Principles and current strategies for targeting autophagy for cancer treatment.. Clin Cancer Res.

[17] Guertin DA, Sabatini DM (2007). Defining the role of mTOR in cancer.. Cancer Cell.

[18] Jung CH, Jun CB, Ro SH, Kim YM, Otto NM (2009). ULK-Atg13-FIP200 complexes mediate mTOR signaling to the autophagy machinery.. Mol Biol Cell.

[19] Hosokawa N, Hara T, Kaizuka T, Kishi C, Takamura A (2009). Nutrient-dependent mTORC1 association with the ULK1-Atg13–FIP200 complex required for autophagy.. Mol Biol Cell.

[20] Hara T, Takamura A, Kishi C, Iemura S, Natsume T (2008). FIP200, a ULK-interacting protein, is required for autophagosome formation in mammalian cells.. J Cell Biol.

[21] Ganley IG, Lam du H, Wang J, Ding X, Chen S (2009). ULK1.ATG13.FIP200 complex mediates mTOR signaling and is essential for autophagy.. J Biol Chem.

[22] Motzer RJ, Escudier B, Oudard S, Hutson TE, Porta C (2008). Efficacy of everolimus in advanced renal cell carcinoma: a double-blind, randomised, placebo-controlled phase III trial.. Lancet.

[23] Hudes GR (2009). Targeting mTOR in renal cell carcinoma.. Cancer.

[24] Amaravadi RK, Yu D, Lum JJ, Bui T, Christophorou MA (2007). Autophagy inhibition enhances therapy-induced apoptosis in a Myc-induced model of lymphoma.. J Clin Invest.

[25] Maclean KH, Dorsey FC, Cleveland JL, Kastan MB (2008). Targeting lysosomal degradation induces p53-dependent cell death and prevents cancer in mouse models of lymphomagenesis.. J Clin Invest.

[26] Altman B, Jacobs S, Mason E, Michalek R, Macintyre A (2011). Autophagy is essential to suppress cell stress and to allow BCR-Abl-mediated leukemogenesis.. Oncogene.

[27] Carew JS, Nawrocki ST, Kahue CN, Zhang H, Yang C (2007). Targeting autophagy augments the anticancer activity of the histone deacetylase inhibitor SAHA to overcome Bcr-Abl-mediated drug resistance.. Blood.

[28] Fan QW, Cheng C, Hackett C, Feldman M, Houseman BT (2010). Akt and autophagy cooperate to promote survival of drug-resistant glioma.. Sci Signal.

[29] Yang S, Wang X, Contino G, Liesa M, Sahin E (2011). Pancreatic cancers require autophagy for tumor growth.. Genes Dev.

[30] Parkhitko A, Myachina F, Morrison TA, Hindi KM, Auricchio N (2011). Tumorigenesis in tuberous sclerosis complex is autophagy and p62/sequestosome 1 (SQSTM1)-dependent.. Proc Natl Acad Sci U S A.

[31] Yazbeck VY, Buglio D, Georgakis GV, Li Y, Iwado E (2008). Temsirolimus downregulates p21 without altering cyclin D1 expression and induces autophagy and synergizes with vorinostat in mantle cell lymphoma.. Exp Hematol.

[32] Ravikumar B, Vacher C, Berger Z, Davies JE, Luo S (2004). Inhibition of mTOR induces autophagy and reduces toxicity of polyglutamine expansions in fly and mouse models of Huntington disease.. Nat Genet.

[33] Lock R, Roy S, Kenific CM, Su JS, Salas E (2011). Autophagy facilitates glycolysis during Ras-mediated oncogenic transformation.. Mol Biol Cell.

[34] Galluzzi L, Kroemer G (2008). Necroptosis: a specialized pathway of programmed necrosis.. Cell.

[35] Davis CW, Hawkins BJ, Ramasamy S, Irrinki KM, Cameron BA (2010). Nitration of the mitochondrial complex I subunit NDUFB8 elicits RIP1- and RIP3-mediated necrosis.. Free Radic Biol Med.

[36] Degterev A, Hitomi J, Germscheid M, Ch'en IL, Korkina O (2008). Identification of RIP1 kinase as a specific cellular target of necrostatins.. Nat Chem Biol.

[37] Yuan J, Kroemer G (2010). Alternative cell death mechanisms in development and beyond.. Genes Dev.

[38] Vanlangenakker N, Vanden Berghe T, Vandenabeele P (2012). Many stimuli pull the necrotic trigger, an overview.. Cell Death Differ.

[39] Yamamoto A, Tagawa Y, Yoshimori T, Moriyama Y, Masaki R (1998). Bafilomycin A1 prevents maturation of autophagic vacuoles by inhibiting fusion between autophagosomes and lysosomes in rat hepatoma cell line, H-4-II-E cells.. Cell Struct Funct.

[40] Edinger AL, Linardic CM, Chiang GG, Thompson CB, Abraham RT (2003). Differential effects of rapamycin on mammalian target of rapamycin signaling functions in mammalian cells.. Cancer Res.

[41] Yu K, Shi C, Toral-Barza L, Lucas J, Shor B (2010). Beyond rapalog therapy: preclinical pharmacology and antitumor activity of WYE-125132, an ATP-competitive and specific inhibitor of mTORC1 and mTORC2.. Cancer Res.

[42] Wu M, Neilson A, Swift AL, Moran R, Tamagnine J (2007). Multiparameter metabolic analysis reveals a close link between attenuated mitochondrial bioenergetic function and enhanced glycolysis dependency in human tumor cells.. Am J Physiol Cell Physiol.

[43] Youle RJ, Narendra DP (2011). Mechanisms of mitophagy.. Nat Rev Mol Cell Biol.

[44] Narendra D, Tanaka A, Suen DF, Youle RJ (2008). Parkin is recruited selectively to impaired mitochondria and promotes their autophagy.. J Cell Biol.

[45] Vives-Bauza C, Zhou C, Huang Y, Cui M, de Vries RL (2010). PINK1-dependent recruitment of Parkin to mitochondria in mitophagy.. Proc Natl Acad Sci U S A.

[46] Lau A, Villeneuve NF, Sun Z, Wong PK, Zhang DD (2008). Dual roles of Nrf2 in cancer.. Pharmacol Res.

[47] Sun Z, Zhang S, Chan JY, Zhang DD (2007). Keap1 controls postinduction repression of the Nrf2-mediated antioxidant response by escorting nuclear export of Nrf2.. Mol Cell Biol.

[48] Komatsu M, Kurokawa H, Waguri S, Taguchi K, Kobayashi A (2010). The selective autophagy substrate p62 activates the stress responsive transcription factor Nrf2 through inactivation of Keap1.. Nat Cell Biol.

[49] Lau A, Wang XJ, Zhao F, Villeneuve NF, Wu T (2010). A noncanonical mechanism of Nrf2 activation by autophagy deficiency: direct interaction between Keap1 and p62.. Mol Cell Biol.

[50] Salazar M, Rojo AI, Velasco D, de Sagarra RM, Cuadrado A (2006). Glycogen synthase kinase-3beta inhibits the xenobiotic and antioxidant cell response by direct phosphorylation and nuclear exclusion of the transcription factor Nrf2.. J Biol Chem.

[51] Merchan JR, Pitot HC, Qin R, Liu G, Fitch TR (2009). Phase I/II trial of CCI 779 and bevacizumab in advanced renal cell carcinoma (RCC): Safety and activity in RTKI refractory RCC patients.. ??J Clin Oncol (Meeting Abstracts).

[52] Efeyan A, Sabatini DM (2010). mTOR and cancer: many loops in one pathway.. Curr Opin Cell Biol.

[53] Klionsky DJ, Abeliovich H, Agostinis P, Agrawal DK, Aliev G (2008). Guidelines for the use and interpretation of assays for monitoring autophagy in higher eukaryotes.. Autophagy.

[54] Ding WX, Ni HM, Gao W, Chen X, Kang JH (2009). Oncogenic transformation confers a selective susceptibility to the combined suppression of the proteasome and autophagy.. Mol Cancer Ther.

[55] Bellodi C, Lidonnici MR, Hamilton A, Helgason GV, Soliera AR (2009). Targeting autophagy potentiates tyrosine kinase inhibitor-induced cell death in Philadelphia chromosome-positive cells, including primary CML stem cells.. J Clin Invest.

[56] Degtyarev M, De Maziere A, Orr C, Lin J, Lee BB (2008). Akt inhibition promotes autophagy and sensitizes PTEN-null tumors to lysosomotropic agents.. J Cell Biol.

[57] Turcotte S, Chan DA, Sutphin PD, Hay MP, Denny WA (2008). A molecule targeting VHL-deficient renal cell carcinoma that induces autophagy.. Cancer Cell.

[58] Turcotte S, Sutphin PD, Giaccia AJ (2008). Targeted therapy for the loss of von Hippel-Lindau in renal cell carcinoma: a novel molecule that induces autophagic cell death.. Autophagy.

[59] Boya P, Gonzalez-Polo RA, Casares N, Perfettini JL, Dessen P (2005). Inhibition of macroautophagy triggers apoptosis.. Mol Cell Biol.

[60] Kaiser WJ, Upton JW, Long AB, Livingston-Rosanoff D, Daley-Bauer LP (2011). RIP3 mediates the embryonic lethality of caspase-8-deficient mice.. Nature.

[61] Oberst A, Dillon CP, Weinlich R, McCormick LL, Fitzgerald P (2011). Catalytic activity of the caspase-8-FLIP(L) complex inhibits RIPK3-dependent necrosis.. Nature.

[62] Zhang H, Zhou X, McQuade T, Li J, Chan FK (2011). Functional complementation between FADD and RIP1 in embryos and lymphocytes.. Nature.

[63] Cho YS, Challa S, Moquin D, Genga R, Ray TD (2009). Phosphorylation-driven assembly of the RIP1-RIP3 complex regulates programmed necrosis and virus-induced inflammation.. Cell.

[64] Upton JW, Kaiser WJ, Mocarski ES (2010). Virus inhibition of RIP3-dependent necrosis.. Cell Host Microbe.

[65] Galluzzi L, Vanden Berghe T, Vanlangenakker N, Buettner S, Eisenberg T (2011). Programmed necrosis from molecules to health and disease.. Int Rev Cell Mol Biol.

[66] Horita H, Frankel AE, Thorburn A (2008). Acute myeloid leukemia-targeted toxin activates both apoptotic and necroptotic death mechanisms.. PLoS One.

[67] Hu X, Han W, Li L (2007). Targeting the weak point of cancer by induction of necroptosis.. Autophagy.

[68] Tse C, Shoemaker AR, Adickes J, Anderson MG, Chen J (2008). ABT-263: a potent and orally bioavailable Bcl-2 family inhibitor.. Cancer Res.

[69] Shibata T, Ohta T, Tong KI, Kokubu A, Odogawa R (2008). Cancer related mutations in NRF2 impair its recognition by Keap1-Cul3 E3 ligase and promote malignancy.. Proc Natl Acad Sci U S A.

[70] Anglard P, Trahan E, Liu S, Latif F, Merino MJ (1992). Molecular and cellular characterization of human renal cell carcinoma cell lines.. Cancer Res.

[71] Grossman HB, Wedemeyer G, Ren LQ (1985). Human renal carcinoma: characterization of five new cell lines.. J Surg Oncol.

[72] Nelson DA, Tan TT, Rabson AB, Anderson D, Degenhardt K (2004). Hypoxia and defective apoptosis drive genomic instability and tumorigenesis.. Genes Dev.

[73] Root DE, Hacohen N, Hahn WC, Lander ES, Sabatini DM (2006). Genome-scale loss-of-function screening with a lentiviral RNAi library.. Nat Methods.

[74] Munger J, Bajad SU, Coller HA, Shenk T, Rabinowitz JD (2006). Dynamics of the cellular metabolome during human cytomegalovirus infection.. PLoS Pathog.

