# Supplementary figures and images for: Autophagy Suppresses RIP Kinase-Dependent Necrosis Enabling Survival to mTOR Inhibition
Source: PLoS One. 2012 Jul 26;7(7):e41831. doi: 10.1371/journal.pone.0041831 (PMC3406086; doi:10.1371/journal.pone.0041831)

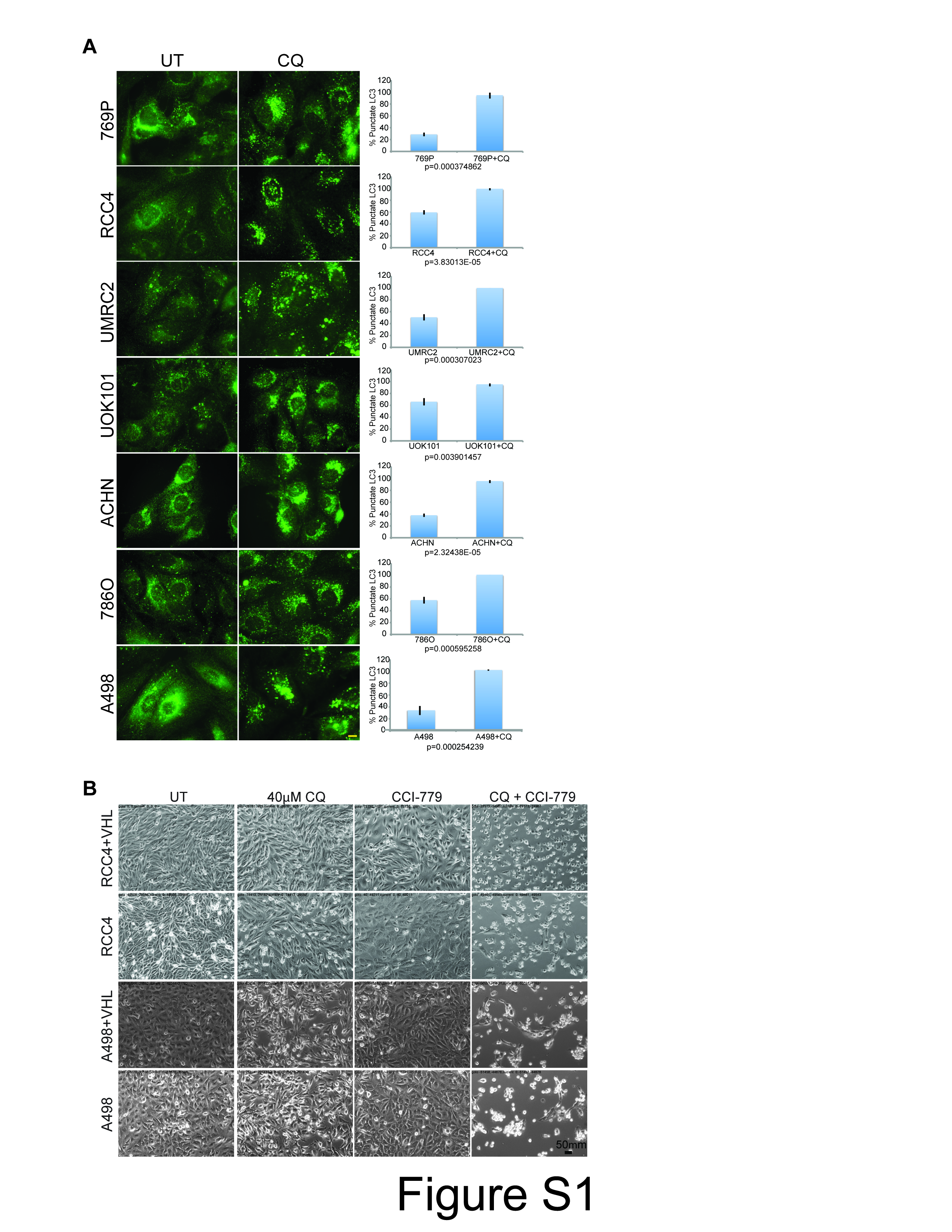

Supplement: Figure S1 — Renal carcinoma cell lines have high basal autophagy and are sensitive to the combination of CQ and CCI-779. (Related to Figure 1) (A) Endogenous LC3 immunostaining in human RCC cell lines (hRCCs). hRCCs were left untreated or treated with 40 µM CQ for 3 hours. Levels of autophagy were quantitated by counting the % of cells with more than 20 LC3 puncta as positive. All hRCCs have high basal autophagy and show 100% punctation with treated with CQ indicating high autophagic flux. Scale bar = 10 µm. (B) Representative stills from Movies S1, S2, S3, S4, S5, S6, S7, S8, S9, S10, S11, S12, S13, S14, S15, S16. RCC4 and A498, with and without VHL, were incubated with 40 µM CCI-779, 40 µM CQ or combination of CQ and CCI-779 for 18 hours. Combination of CQ and CCI-779 dramatically induced cell death in RCC cells. (TIF) [file pone.0041831.s001.tif]

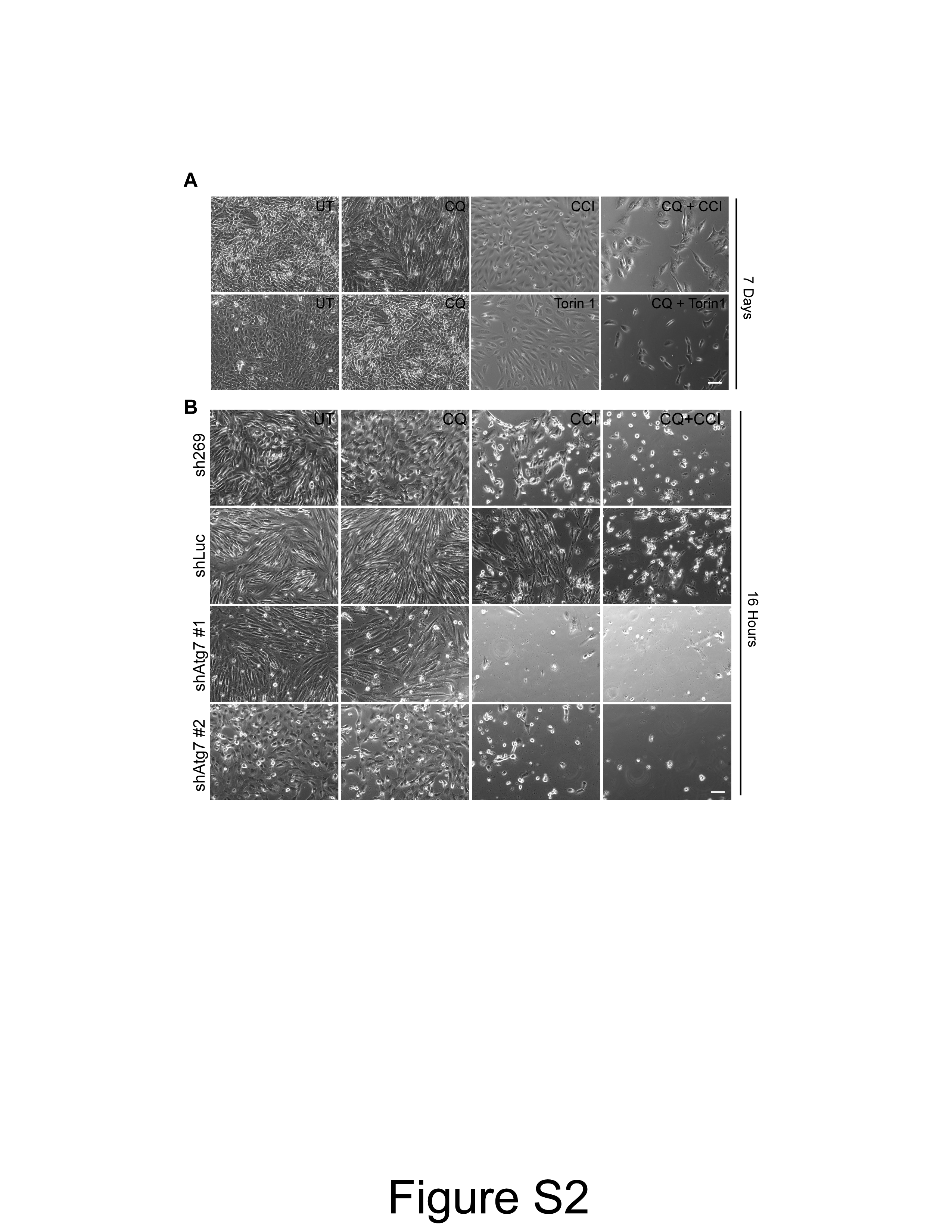

Supplement: Figure S2 — Autophagy and mTOR inhibition induces cell death. (Related to Figure 2 ) (A) Representative images of Fig. 2A and B after 7 days of drug incubation demonstrating toxicity of 20 µM CQ and 20 µM CCI-779 or 20 µM CQ and 250 nM Torin1. Scale bar = 50 microns. (B) Representative images of Fig. 2D showing RCC4 cells with control knockdown (shRfp, shLuc) or knockdown of Atg7 (shAtg7#1,2). Atg7 knockdown sensitized RCC4 to 40 µM CCI-779. Scale bar = 50 microns. (TIF) [file pone.0041831.s002.tif]

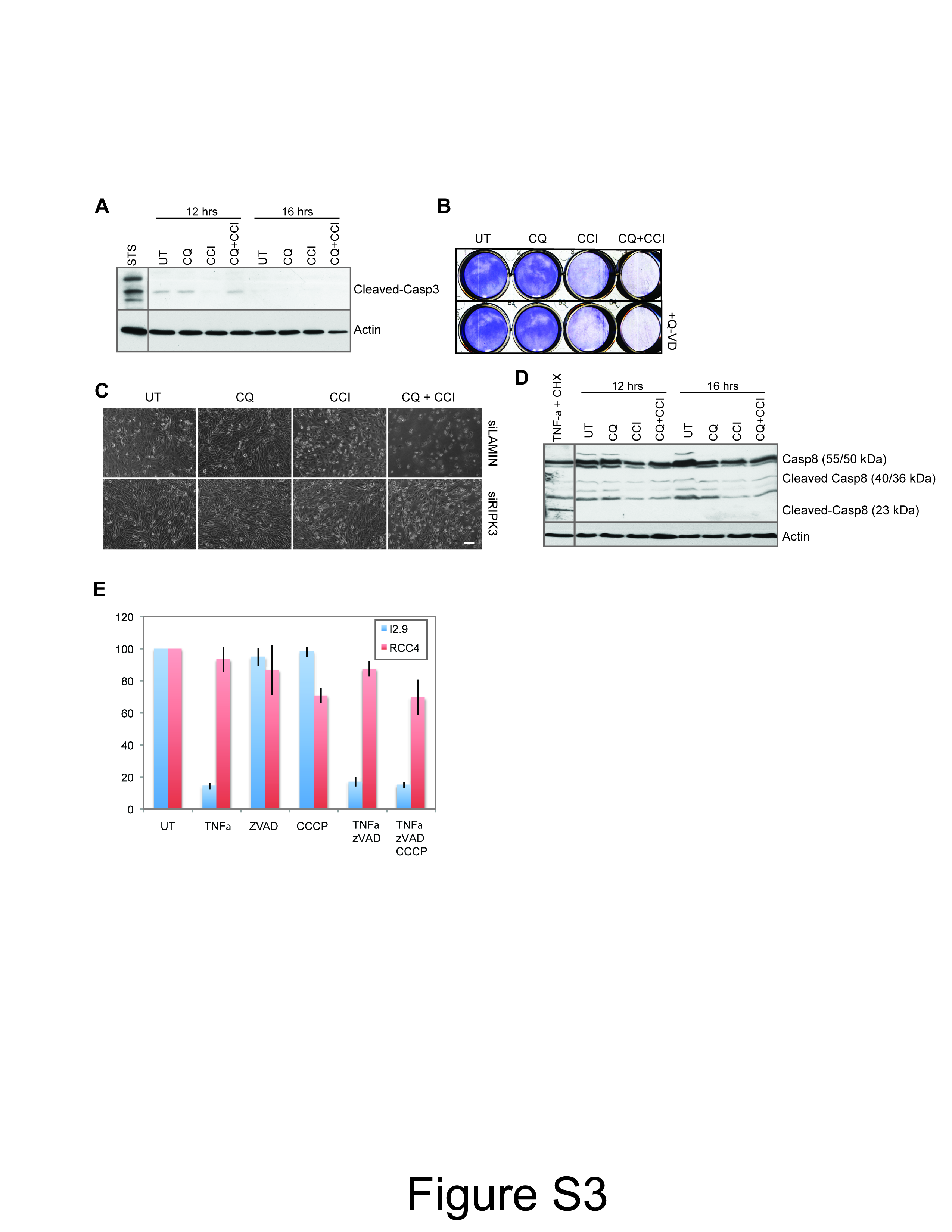

Supplement: Figure S3 — Autophagy and mTOR inhibition induces necroptosis. (Related to Figure 3 ) (A) Western blot for cleaved caspase-3 in RCC4 treated with CQ, CCI-779 and combination of 40 µM CQ and 40 µM CCI-779 at 16 hrs. As a control RCC4 cells were treated with 4 µM staurosporine for 10 hours. (B) Clonogenic survival assay showing that the pan-caspase inhibitor Q-VD-OPh does not rescue cell death induced by CQ and CCI. RCC4 was pretreated with 20 µM Q-VD-OPh for 1 hour followed by the addition of 40 µM CQ and 40 µM CCI to the media. After an 18 hour drug treatment media was changed to normal growth media and cells were allowed to recover for 5 days. (C) Representative images of Fig. 3B. Knockdown of RIP3K in RCC4 rescues cell death induced by 40 µM CQ and 40 µM CCI as compared to Lamin control knockdown. Scale bar = 50 µM. (D) Western blot showing that RCC4 cells do not cleave Caspase-8 in response to 40 µM CQ and 40 µM CCI in comparison to control treated with 100 nM TNF-α and 10 µg/ml cycloheximide. (E) RCC4 and I2.1 (Fadd−/− Jurkat cells) were treated with 100 nM TNF-α and 50 µM Z-VAD-FMK. TNF-α alone induced necroptosis in I2.1 cells. RCC4 remained viable even in the presence of Z-VAD-FMK. (TIF) [file pone.0041831.s003.tif]

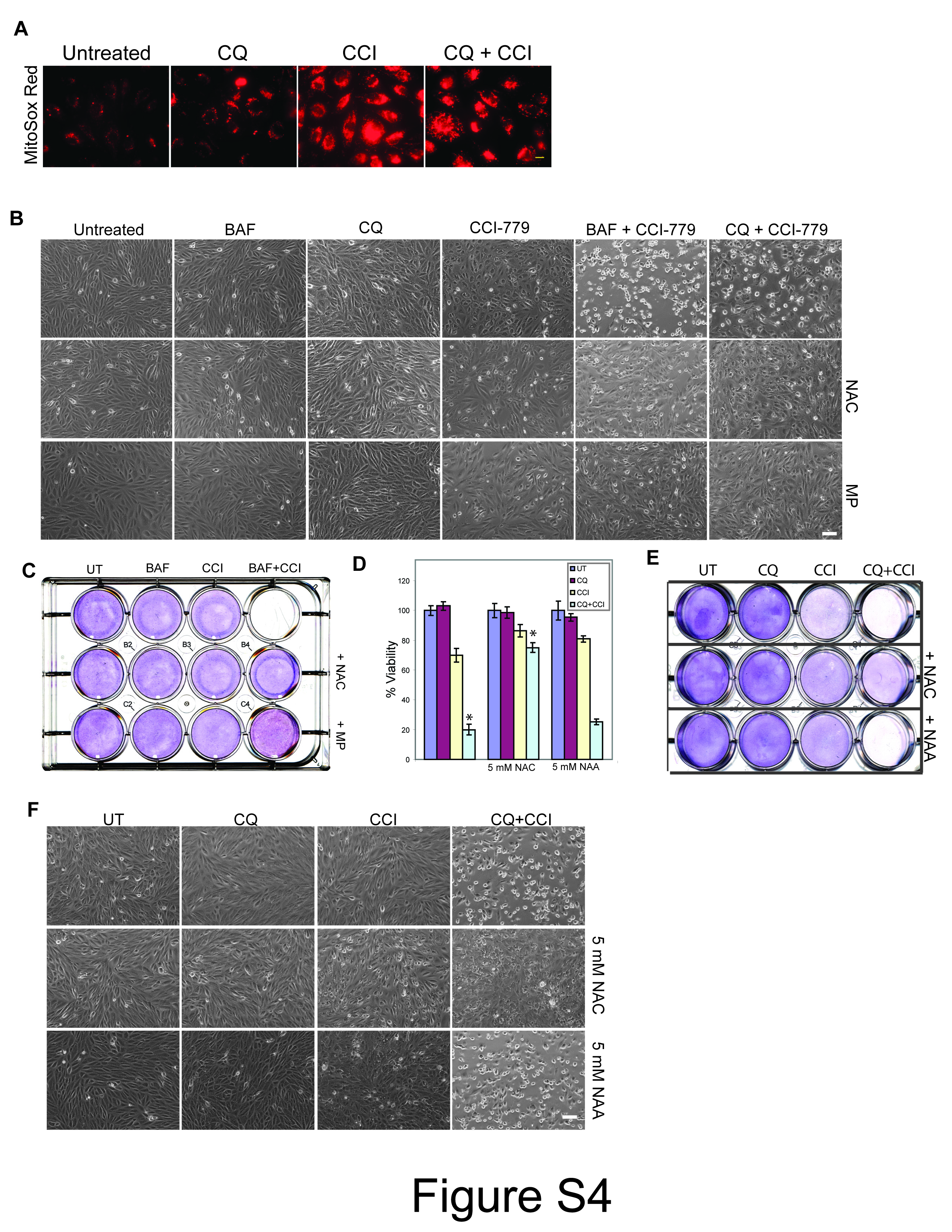

Supplement: Figure S4 — Mitochondrial ROS production causes cell death that is rescued by NAC and methyl-pyruvate. (Related to Figure 4 ) (A) MitoSox Red superoxide indicator staining of RCC4 cells. 40 µM CCI-779 and combination of 40 µM CQ and 40 µM CCI-779 had high levels of ROS as indicated by MitoSox Red fluorescence after a 6 hour drug incubation. Scale bar = 10 µM. (B) Representative images of Fig. 4B. RCC4 was treated with 4 nM Bafilomycin A1, 40 µM CQ, 40 µM CCI-779 or combination of Bafilomycin A1 and CCI-779 or CQ and CCI-779. The combination of Bafilomycin A1 (BAF) or CQ with CCI-779 induces cell death that is rescue with 5 mM n-acetyl-cysteine (NAC) or 8 mM methyl-pyruvate (MP). Scale bar = 50 µM. (C) Clonogenic survival assay. Combination of BAF and CCI-779 induces cell death that is rescued by NAC and MP. (D) Cell death induced by combination of CQ and CCI-779 is rescued by NAC but not n-acetyl-alanine (NAA). (*p = 2.01×10^−4^) (E) Clonogenic assay of treatment shown in D. (F). Representative photos of D. Scale Bar = 50 µM. (TIF) [file pone.0041831.s004.tif]

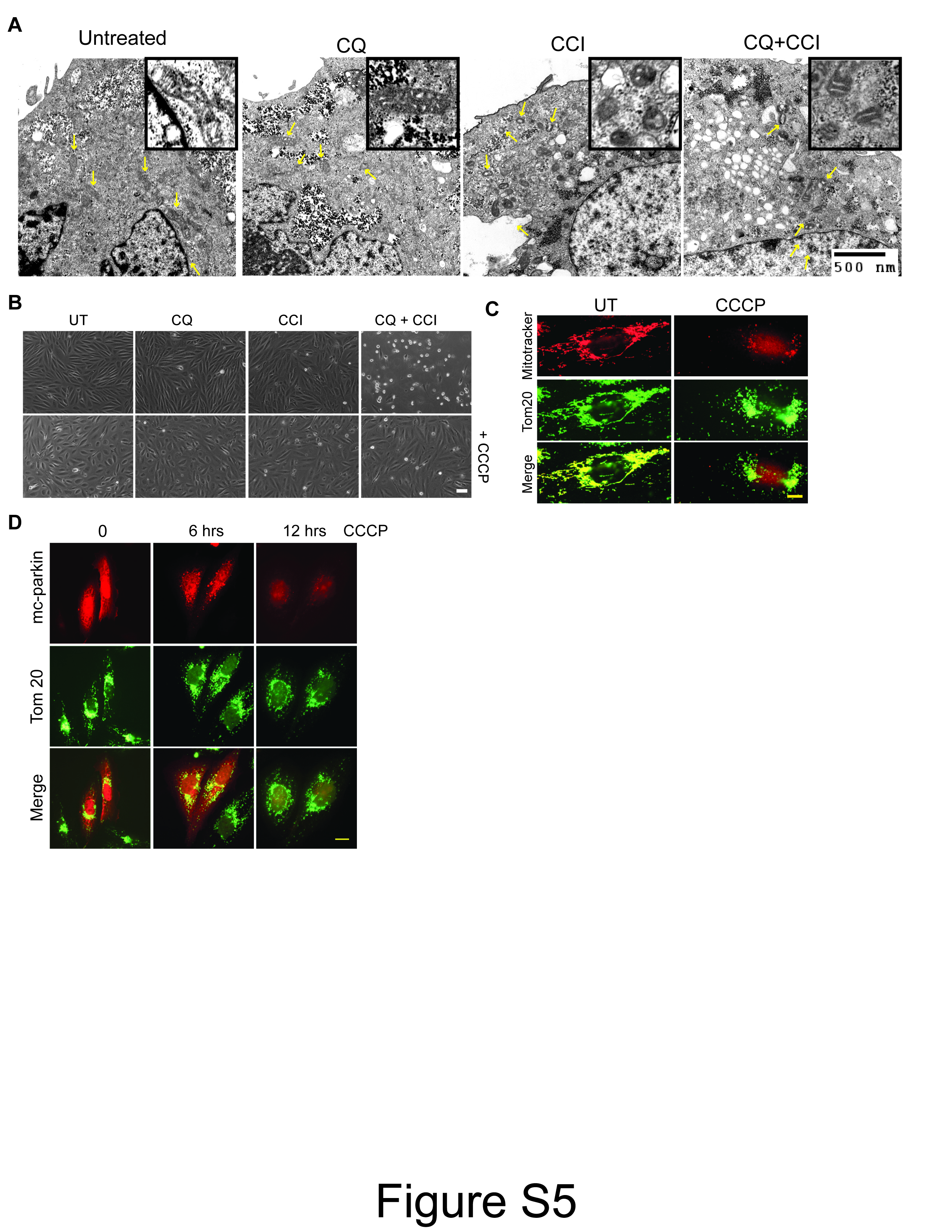

Supplement: Figure S5 — Mitochondrial are required for necroptosis. (Related to Figure 4 ) (A) Electron micrographs of RCC4 treated with 40 µM CQ, 40 µM CCI-779, or combination of CQ and CCI-779. Mitochondrial appear fragmented with CCI-779 or combination of CQ and CCI-779. (B) Representative images of Fig. 4I. 10 µM CCCP rescued cell death induced by the combination of CQ and CCI-779 in RCC4 cells. Scale Bar = 50 µM. (C) Mitochondrial potential assay showing loss of mitotracker red and membrane potential as compared to total mitochondria (Tom20) after a 6 hour incubation with 10 µM CCCP. Scale Bar = 10 µM. (D) CCCP caused m-cherry-Parkin to translocate to the mitochondria. Scale Bar = 10 µM. (TIF) [file pone.0041831.s005.tif]

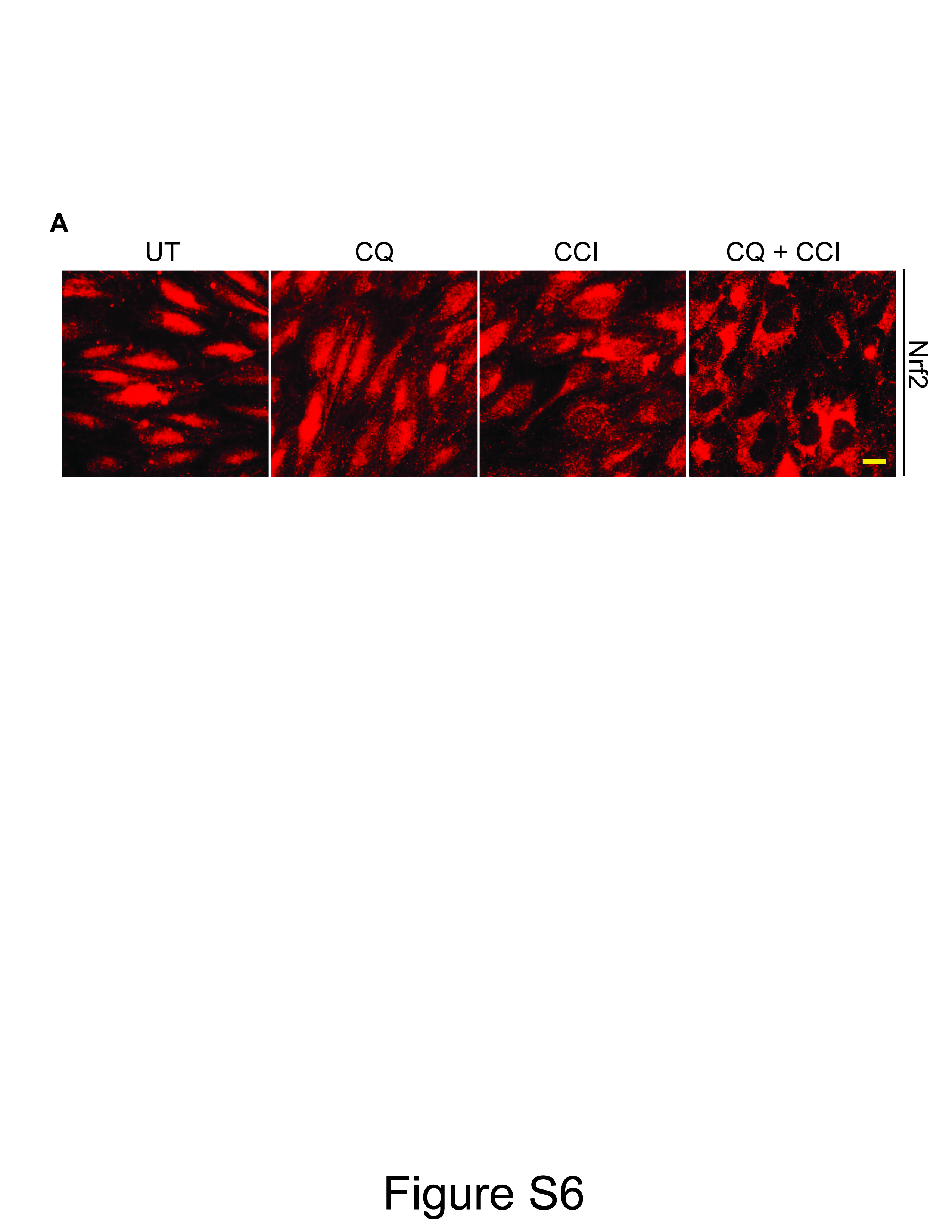

Supplement: Figure S6 — CCI-779 blocks nuclear translocation if Nrf2. (Related to Figure 5 ) Endogenous staining of Nrf2 in RCC4 cells after a 6 hour drug incubation. Nrf2 is predominately nuclear when left untreated or with 40 µM CQ. Nrf2 is excluded from the nuclease with 40 µM CCI-779 or combination of CQ and CCI-779. Scale bar = 10 µM. (TIF) [file pone.0041831.s006.tif]
